# Imprime PGG Enhances Anti-Tumor Effects of Tumor-Targeting, Anti-Angiogenic, and Immune Checkpoint Inhibitor Antibodies

**DOI:** 10.3389/fonc.2022.869078

**Published:** 2022-05-26

**Authors:** Anissa S. H. Chan, Takashi O. Kangas, Xiaohong Qiu, Mark T. Uhlik, Ross B. Fulton, Nadine R. Ottoson, Keith B. Gorden, Yumi Yokoyama, Michael E. Danielson, Trinda M. Jevne, Kyle S. Michel, Jeremy R. Graff, Nandita Bose

**Affiliations:** ^1^ HiberCell Inc., Roseville, MN, United States; ^2^ Biothera Pharmaceuticals Inc., Eagan, MN, United States

**Keywords:** Imprime PGG, pathogen-associated molecular pattern, Dectin-1, cancer immunotherapy, immune checkpoint inhibitors

## Abstract

Imprime PGG (Imprime) is in late-stage clinical development as a combinatorial agent with several therapeutic modalities. Here we present pre-clinical mechanistic data supportive of Imprime, a soluble yeast β-1,3/1,6-glucan pathogen-associated molecular pattern able to prime innate immune cells in a Dectin-1dependent manner. In tumor-free mice, Imprime evoked broad innate immune responses (type I interferon signature, mobilization of myeloid cells, dendritic cell and monocyte/macrophage expression of co-stimulatory ligands like CD86, and activation of natural killer cells). Imprime-mediated activation of myeloid cells also resulted in functional priming of antigen-specific CD8 T cell response. In tumor-bearing mice, Imprime monotherapy further resulted in activation of systemic and tumor infiltrating macrophages and enhanced cytotoxic CD8 T cell trafficking. Imprime enhanced the anti-tumor activity of several combinatorial agents in mouse cancer models; anti-tyrosinase-related protein 1 antibody in B16F10 melanoma experimental lung metastasis model, anti-vascular endothelial growth factor receptor 2 antibody in H1299 and H441 lung cancer, and anti-programmed cell death protein 1 antibody in MC38 colon cancer models. Mechanistically, combining Imprime with these combinatorial therapeutic agents elicited enhanced innate immune activation, supporting immunological synergy. Finally, Imprime treatment induced similar *in vitro* phenotypic and functional activation of human innate immune cells. Collectively, these data demonstrate Imprime’s potential to orchestrate a broad, yet coordinated, anti-cancer immune response and complement existing cancer immunotherapies.

## 1 Introduction

Immunotherapy has emerged as the frontline treatment of several cancer types, albeit in a fraction of cancer patients. Immune checkpoint inhibitor (ICI) therapy provides compelling clinical benefit primarily in patients with pre-existing anti-tumor T cell responses. A majority of the patients either demonstrate primary resistance and fail to respond to immune checkpoint blockade or develop acquired resistance and relapse after showing an initial response (1). Highly specialized adoptive cell therapies such as tumor infiltrating lymphocyte therapy, engineered T cell receptor therapy, and chimeric antigen receptor T cell therapy have shown unprecedented efficacy but only in a subset of patients in limited cancer types (2, 3). Additionally, some of these therapies have also been associated with diverse and serious immune-related adverse effects that further limit their clinical benefits. It is therefore critical to develop rational combination strategies that improve efficacy and overcome resistance without exacerbating the immune-related side effects.

While the majority of cancer immunotherapies focus on harnessing the cytotoxic T cell response, the role of innate immune effector function has been critical to the success of several cancer therapies. Tumor-targeting antibodies (Abs) block oncogenic signaling but can also opsonize cancer cells to induce innate immune cytotoxic mechanisms such as Ab-dependent cellular cytotoxicity, (ADCC), complement-dependent cytotoxicity, and Ab-dependent cellular phagocytosis by monocytes/macrophages, natural killer (NK) cells, and neutrophils. Anti-angiogenics block vascular endothelial growth factor (VEGF) signaling, and in doing so, foster T cell immunity by allowing dendritic cell (DC) maturation and repolarization of M2 to M1 macrophages (4). Furthermore, therapies that propagate the anti-tumor T cell-based cancer immunity cycle, like ICIs, engage the innate immune system by priming DCs to present to antigen-specific T cells and by reorienting the immunosuppressive myeloid cells in the tumor immune microenvironment to allow for trafficking and activation of the cytotoxic T cells (5). As such, it is logical to conclude that targeting innate immunity to provide broader activation of the immune system may be the foundation upon which rational synergistic immunotherapy combinations could be conceptualized. However, clinical development of such combinations has proven to be a challenge since innate immune modulators have the potential to induce acute systemic inflammation. Short half-life, narrow therapeutic index, and suboptimal delivery to the target site have been some of the hurdles to the systemic administration of synthetic pathogen-associated molecular pattern (PAMP)-mimics, including Toll-like receptor (TLR) agonists (6–9).

Imprime PGG (Imprime) is an intravenously (i.v.) administered yeast β-1,3/1,6-glucan (BTH1677) PAMP. Imprime has now been administered to more than 500 cancer patients in a series of clinical trials in combination with tumor-targeting Abs, anti-angiogenic Abs, and most recently, with ICIs (10–14). In each of these trials, Imprime and the combinatorial agent showed improved clinical responses compared to that observed with the combinatorial agent in either the historical trials or a randomized setting. In addition to the encouraging clinical results from several trials, Imprime, has been shown to have a favorable safety profile as monotherapy in healthy volunteer trials, and as combination therapies in cancer trials in spite of being a systemically administered innate immune modulator (10, 12, 13, 15, 16).

Presented herein are the pre-clinical data providing the immunological basis for these promising clinical trials. These data show that Imprime elicits 1) increased cytotoxic responses from innate immune cells, specifically NK cell-mediated tumor killing; 2) enhanced maturation and antigen presentation function of DCs; 3) innate immune reprogramming to repolarize the normally suppressive macrophages that dominate the tumor immune microenvironment; and 4) a robust infiltration of activated CD8 T cells in the tumor. *In vivo* preclinical mouse tumor models and *ex vivo* human whole blood (WB) experimental systems were used to demonstrate tumor growth inhibition and immunomodulatory mechanisms of Imprime alone as well as in combination with various therapeutic agents. These results showed significant synergy in efficacy with multiple combination agents: Imprime, in combination with TA99, an Ab targeting tumor tyrosinase-related protein 1 (Tyrp1) when tested in the B16F10 experimental metastasis melanoma model, Imprime with DC-101, an anti-angiogenic Ab targeting VEGF receptor (VEGFR) 2 in the H441 and H1299 NSCLC cancer models, and finally, Imprime with anti-PD-1 Ab in the MC38 colon cancer model. Collectively, these pre-clinical data, armed with the promising signals of clinical benefit, support the ongoing clinical development of Imprime as a combination therapy in multiple cancer indications.

## 2 Materials and Methods

### 2.1 Imprime PGG

Imprime PGG (Imprime; BTH1677 or β(1,6)-[poly-(1,3)-D-glucopyranosyl]-poly-β-(1,3)-D-glucopyranose; Hibercell Inc.) is a water-soluble β-1,3/1,6 glucan purified from the cell wall of a proprietary, non-recombinant strain of *Saccharomyces cerevisiae*. It is comprised of a D-glucose β-1,3 linked backbone with β-1,6 D-glucose side chains and has an average molecular weight of 150 kDa as described previously (17).

### 2.2 *In Vivo* Mouse Experiments

#### 2.2.1 Mice

Female C57BL/6 mice were purchased from Charles River Laboratories. OT-1 T cell receptor transgenic mice were a kind gift from Drs. Kris Hogquist and Stephen Jameson (University of Minnesota, Minneapolis, MN). Dectin-1 knockout (KO) (B6.129S6-*Clec7a^tm1Gdb^*/J) mice were a kind gift from Dr. Dan Kaplan (University of Minnesota), and major histocompatibility complex (MHC) class I-deficient transporter for antigen presentation (TAP) KO (TAPKO; B6.129S2-*Tap1^tm1Arp^*/J) mice were purchased from the Jackson Laboratory. All mice were maintained in specific-pathogen-free conditions.

#### 2.2.2 Gene Expression by QuantiGene Plex

Mice were injected i.v. with 1.2 mg Imprime or phosphate-buffered saline (PBS; vehicle). Sixteen hours later, mice were euthanized, and spleens and skin-draining lymph nodes (sdLNs) were harvested and stored in 1 ml RNAlater Solution (Thermo Fisher Scientific) at -20°C until processed. To isolate RNA, tissues were disrupted in RLT buffer (Qiagen) using biomasher tubes (Kimble), and lysates were then passed through QIAshredder (Qiagen). Standard procedures were followed using Qiagen RNeasy Mini Kits to obtain purified RNA, and concentrations were determined using an Epoch microplate spectrophotometer using Gen5 2.0 software. QuantiGene Plex (Thermo Fisher Scientific) was used for transcriptional analysis. Five hundred to 1000 ng purified RNA were used for input, and the QuantiGene Plex protocol was followed for all subsequent steps. Samples were run on a Luminex xMAP 200 using xPonent 3.1 software (Thermo Fisher Scientific). Data were exported as mean fluorescence intensity (MFI) values. To calculate fold changes in gene expression, background MFI values were subtracted from experimental samples. Within each sample, MFI values were then normalized to the geometric mean of the loading controls peptidylprolyl isomerase B, hypoxanthine guanine phosphoribosyl transferase 1, and TATA box binding protein. Within each mouse strain (i.e., wild type [WT] versus Dectin-1 KO), fold change for each gene was calculated by dividing each sample by the average of the vehicle-treated samples.

#### 2.2.3 *In Vivo* Detection of Cytokines and Chemokines

At various times following Imprime treatment or lipopolysaccharide (LPS) injection (i.v., 2 µg), serum, spleens, and sdLNs (pooled inguinal, axillary, and brachial lymph nodes) were harvested and sdLNs were appropriately weighed. Tissues were then manually disrupted, and particulate cellular debris was pelleted in a refrigerated table-top centrifuge at maximum speed. The clarified supernatant was collected and stored at -80°C until analysis. Cytokine and chemokine proteins were detected using mouse pre-designed ProcartaPlex panels (Thermo Fisher Scientific) according to the manufacturer’s instructions. Samples were run on a Luminex xMAP 200 using Luminex xPonent 3.1 software and analyzed using Milliplex Analyst software (Merck Millipore). Data were adjusted for dilution factors and normalized per 10 mg tissue.

#### 2.2.4 Flow Cytometry

Single cell suspensions from the harvested sdLNs and spleen were prepared according to standard procedures. Cells were washed with washing buffer (PBS containing 2% fetal calf serum and 0.02% sodium azide) and then stained with a master mix containing Abs for cell surface markers (listed in **Supplementary Table 1**). For intracellular staining of transcription factors and cytokines, cells were stained using Foxp3/Transcription Factor Staining Buffer kit (Tonbo Biosciences) and BD Fix/perm buffer kit (BD Biosciences), respectively, according to the vendor’s instruction. For binding experiments, Imprime-bound to mouse and human immune cells were detected by anti-glucan rabbit polyclonal primary antibody and BfD IV mouse IgM monoclonal antibody respectively. Samples were run on the BD Fortessa flow cytometer (BD Biosciences), and data were analyzed using FlowJo version 10 (FlowJo, LLC). Gating strategies for identification of immune cell subsets are provided in **Supplementary Figure 1**.

#### 2.2.5 NK Killing Assay

C57BL/6 mice were injected intraperitoneally (i.p.) with NK1.1 Ab (PK136) or isotype control. The next day, mice were treated with vehicle, Imprime, or Poly(I:C) (2 µg). Twenty-four hours later, mice were injected i.v. with a 1:1 ratio of 5 x10^6^ carboxyfluorescein succinimidyl ester (CFSE; Tonbo Bioscience)-labeled WT to Celltrace™ violet (Thermo Fisher Scientific)-labeled TAPKO splenic cells. Recipient mice splenic cells were harvested after overnight incubation, and single cells were processed and stained for flow cytometry. To evaluate the role of Dectin-1 and interferon (IFN), Dectin-1 KO mice and depletion with an anti-IFN-α/β receptor (IFNAR) Ab were also used in the NK killing assay.

#### 2.2.6 Ovalbumin (OVA) Antigen-Specific CD8 T Cell Activation

To assay the antigen-specific CD8 T cell response, 10^5^ OT-1 cells harvested from OT-1 T cell receptor transgenic mice expressing the congenic marker CD90.1 were adoptively transferred to naïve C57BL/6 mice and after 24 hours, mice were immunized i.v. with OVA_257-264_/H-2K^b^ peptide and either PBS, Imprime, or LPS. Peripheral blood was harvested 5 days later, whereas spleens and sdLNs were harvested 7 days later to assay for OT-1 cell expansion, activation, and cytokine production by flow cytometry.

### 2.3 Assessment of Tumor Growth and Immune Activation in Mouse Tumor Models

#### 2.3.1 MC38 Syngeneic Tumor Model – Tumor Growth

The MC38 cell line was a gift from Michael R. Shurin (University of Pittsburgh Medical Center, Pittsburgh, PA). To establish tumors in mice, MC38 cells cultured in complete RPMI medium (Complete Medium supplemented with 10% fetal calf serum, L-glutamine, and penicillin/streptomycin) were harvested, washed, and resuspended in cold PBS. Five x 10^5^ cells were injected subcutaneously into the right dorsal flanks of shaved mice in a volume of 100 µl. When tumors averaged 50-100 mm^3^ in volume (consistently for 7 days at 50 mm^3^), mice were randomly distributed so that the median and standard deviation in tumor volumes were similar between treatment groups. Tumor volumes were measured using digital calipers and calculated using the following formula: V = (h*w^2^)/2 where w = width and h = height of the shortest and longest dimensions of the tumor, respectively. Mice reached endpoint when tumor volume exceeded 1500 mm^3^; tumor volumes of these mice were then recorded as 1500 mm^3^ until the end of the study. Some tumors developed small involutions and were treated with Collasate (PRN Pharmacal) every other day as long as the tissue remained dry and closed. Mice were euthanized if open tumor ulcerations developed. Tumor-bearing mice were treated twice weekly with either vehicle, 1.2 mg Imprime i.v., 0.1 mg anti-PD-1 i.p. (clone RMP1-14, BioXCell), or a combination of Imprime i.v. + anti-PD-1 i.p. After 14 days of treatment (four treatments in total), tumors were excised for analysis.

#### 2.3.2 MC38 Syngeneic Tumor Model – Immune Monitoring, Flow Cytometry

The spleens and the tumors from the MC38 tumor-bearing mice were excised 7 days after treatment and processed into single cell suspensions according to standard procedures. This single cell suspension was then used to assess phenotypic activation of tumor-associated macrophages (TAMs), splenic macrophages, and splenic monocytic-myeloid-derived suppressor cells (m-MDSCs) by staining with Abs for surface markers (**Supplementary Table 1**) and evaluated by flow cytometry. Tumor necrosis factor (TNF)-α production by TAMs and splenic macrophages in response to overnight LPS stimulation (100 ng/ml) was measured by intracellular staining as previously described.

#### 2.3.3 MC38 Syngeneic Tumor Model – TAM Suppression Assay

TAMs were enriched from the tumor suspension by 40/80 Percoll (GE Healthcare) gradient separation (300 *x g*, 23°C, for 23 minutes), and followed by further enrichment of CD11b^+^ cells using CD11b microbeads (Miltenyi Biotec). The enriched cells were confirmed to be 95% pure for CD11b^+^F4/80^+^ TAM. Splenic T cells were isolated from naïve C57BL/6 mice using the T cell enrichment kit (StemCell Tech), and then labeled with CFSE. One hundred thousand CFSE-labeled splenic T cells were added to wells of a 96-well plate and co-incubated with TAM in the presence of Dynabeads™ mouse T-activator CD3/28 beads per vendor’s instruction (Thermo Fisher Scientific) in a 37°C, 5% CO_2_ incubator for 3 days. T cell proliferation was measured by CFSE dilution to derive division index by flow cytometry. Division Index is defined as the average number of cell divisions that a cell in the original population has undergone.

#### 2.3.4 MC38 Syngeneic Tumor Model – CD8 T Cell Functional Assays

Tumors were excised from euthanized mice on day 14 post-treatments (vehicle, Imprime, anti-PD-1, and Imprime + anti-PD-1), and single cell suspensions were prepared as described above. Tumor infiltrating lymphocytes were enriched from the single cell suspension by a 30/70 Percoll gradient separation (*x* 2000 rpm, 23°C, for 20 minutes). For antigen-specific stimulation, enriched leukocytes were stimulated *ex vivo* with the known antigenic peptide of MC38, p15E (18), for 5 hours, and the intracellular cytokine production in CD8 tumor infiltrating lymphocytes was measured by flow cytometry.

#### 2.3.5 MC38 Syngeneic Tumor Model – Multiplex Immunohistochemistry and Imaging

Mouse tissues were fixed, embedded in paraffin, and 4 mM sections were cut onto slides (University of Minnesota Comparative Pathology Shared Resource). Slides were deparaffinized, rehydrated, and heat-mediated antigen retrieval was conducted with AR6 buffer (PerkinElmer). Slides were blocked with Roche diagnostics Ab diluent (Fisher) and stained with primary Abs for 1 hour at 110 rpm. HRP-conjugated secondary Abs were added for 10 minutes at 110 rpm followed by 10 minutes with Opal detection dye (PerkinElmer). For multiplex staining, this process was repeated starting at the antigen retrieval stage. Multispectral images were captured on the Vectra 3 imaging system (PerkinElmer) and spectral unmixing and cell segmentation was performed using Inform Tissue Finder software (PerkinElmer). Imaging data was converted into .csv files and imported into Flowjo for immune phenotyping. Primary Abs used were rabbit anti-mouse CD3 (clone SP7; Abcam; paired with Opal 520), rabbit anti-mouse CD8 (clone 4SM15; eBioscience; paired with Opal 570), rabbit anti-mouse granzyme B (GrzB; polyclonal ab4059; Abcam; paired with Opal 650), and rabbit anti-mouse Ki67 (clone SP6; Thermo Fisher Scientific; paired with Opal 690). Secondary Abs used were HRP-goat anti-rabbit (Jackson Immunoresearch) and HRP-goat anti-rat (Jackson Immunoresearch).

#### 2.3.6 Syngeneic B16F10 Experimental Lung Metastasis Model

C57BL/6 mice were injected i.v. *via* the tail vein with 1 x 10^5^ B16F10 melanoma cells (ATCC). The anti-Tyrp1 monoclonal Ab TA99 and Imprime were administered i.p. (50 µg/mouse, days 1, 3, 5, 7, 10) and i.v. (1.2 mg/mouse, days 1, 3, 7, 10, 14), respectively. To evaluate the role of CD8 T cells and NK cells, anti-NK1.1, and anti-CD8 mAb were administered at 200 µg on day 1 and then at 100 µg twice weekly for 2 weeks. Mice were euthanized 21 days after tumor challenge in order to count lung metastases. Tumor burden was also assessed by measuring Tyrp1 expression using reverse transcription polymerase chain reaction (RT-PCR). For immunohistochemistry, 21 days after treatment, mice were sacrificed, and lungs were harvested and fixed in neutral-buffered formalin to count B16 metastatic foci. Tumor tissue was prepared, stained, and analyzed by Opal/Vectra multiplex tissue imaging as described above. Rabbit anti-mouse Tyrp1 (clone EPR13063; Abcam) was used to stain B16F10 melanoma cells.

#### 2.3.7 Non-Small Cell Lung Cancer (NSCLC) Xenograft Mouse Models

H441 and H1299 cells were injected subcutaneously into the flanks of nude mice. Once tumors reached 100-150 mm^3^, mice were randomized to ensure similarity in tumor size between treatment groups. The mice received 10 mg/kg of the anti-VEGFR2 Ab DC-101 i.p. and/or 1.2 mg/mouse Imprime i.v. twice weekly for 4 weeks, or control treatment. Tumor volumes were measured using digital calipers. Mice reached endpoint when tumor volume exceeded 1500 mm^3^. Tumor volumes of these mice were then recorded as 1500 mm^3^ until the end of the study. For depletion of macrophages in the H1299 model, 100 µl of clodronate (5 mg/ml) or control liposomes (Encapsulated Nanosciences) were administered i.v. twice weekly. The tumors were excised for RNA analysis and flow cytometric evaluation. Total RNA from the tumor cells was reverse transcribed to first-strand cDNA using a QuantiTect Reverse Transcription Kit (Qiagen). Quantitative RT-PCR was performed using the StepOnePlus Real-Time PCR System (Applied Biosystems) according to the manufacturer’s protocol. The primers and probes were selected from the ABI TaqMan Gene Expression Assay catalog. The expression was normalized to that of *GAPDH*. Fold change was determined by the ΔΔCt method. For flow cytometry, the excised tumors were processed into single cell suspensions and then analyzed by flow cytometry for surface markers and intracellular TNF-α production after stimulation with 100 ng/ml LPS.

### 2.4 *Ex Vivo* Human Studies

#### 2.4.1 *In Vitro* Human WB Cytokine Experiment

Venous anti-coagulated WB (0.5 ml) was obtained from healthy human volunteer subjects. The blood was treated with various test articles for 24 hours. Imprime, TLR-4 agonist LPS (L4516, Sigma), TLR-7/8 agonist resiquimod (4536, Tocris), and stimulator of IFN genes agonist 2’3’-cGAM(PS)_2_ (RP/RS) (tlrl-nacga2srs, *In vivo*gen) were all tested at 10 μg/ml. Cytokine production in the plasma was measured by Luminex using a custom Human ProcartaPlex 21-plex panel (Thermo Fisher Scientific).

#### 2.4.2 NK Cell Cytotoxicity

Human peripheral blood mononuclear cells (PBMCs) were isolated from WB according to standard procedures. One to 2 x 10^6^ PBMCs were incubated with Imprime (25 µg/ml) or vehicle in XVivo15 media (Lonza) with 30% autologous serum for 3 days. PBMCs were washed once with PBS and cultured with K562 human tumor cells (ATCC) in 10% fetal bovine serum with the indicated target:effector ratios for 4 hours. Cytotoxicity was detected by flow cytometry with Live/Dead staining or by CD107a surface staining (LAMP-1 degranulation) on NK cells. Samples were tested in triplicate. Changes in the phenotypic markers on NK cells were also evaluated by flow cytometry.

#### 2.4.3 Monocyte-Derived Macrophage (MDM) Preparation and Suppression Assay

MDM were prepared *in vitro* as described previously (19). Briefly, WB was incubated with 25 µg/ml Imprime or vehicle control at 37°C in a 5% CO_2_ humidified incubator for 2 hours. PBMCs were isolated by ficoll separation followed by CD14^+^ monocyte enrichment using Dynabeads Untouched Human Monocytes kit (Thermo Fisher Scientific). Enriched monocytes (5 x 10^5^ cells/ml) were then cultured in XVivo10 media supplemented with 10% autologous serum and 50 ng/ml recombinant human M-CSF (R&D Systems) for 6 days with medium changed on day 3. Recombinant human interleukin (IL)-4 (R&D Systems) was added to the last 72 hours of incubation. Alternatively, for some experiments, tumor conditioned medium (TCM) was added to the cell culture on day 3 during medium replacement at 70% of the volume. TCM was prepared by culturing BxPC-3 and MiaPaCa (pancreatic cancer cell lines; ATCC) for 48 hours, collecting the culture supernatant, checking VEGF levels by ELISA, and freezing at -70°C until use. For macrophage-T cell co-culture proliferation assay*s*, 5 x 10^4^ CFSE-labeled CD3 T cells were incubated with MDM at a 10:1 ratio in XVivo15 medium for 3-5 days in the presence of plate-bound CD3 and CD28 monoclonal Abs. T cell proliferation was measured by flow cytometry, and division index was calculated.

#### 2.4.4 Imprime Binding to Primary DCs and Preparation of Monocyte-Derived DCs (MoDCs)

*In vitro *Imprime binding in human subjects has been described previously (16, 17). MoDCs were prepared similarly to MDM, except the enriched monocytes were cultured in XVivo15 medium supplemented with 10% human autologous serum in the presence of 50 ng/ml recombinant human GM-CSF (R&D systems) and 50 ng/ml recombinant human IL-4 for 6-7 days. Maturation was induced by LPS (50 ng/ml) and TNF-α (25 ng/ml) for 48-72 hours. For allogeneic mixed lymphocyte reaction, 2.5 x 10^4^ CFSE-labeled CD3 T cells were added to wells of a 96-well plate, incubated with MoDCs at the indicated ratio in a 5% CO_2_ humidified incubator at 37°C for 3-5 days, and proliferation was determined by flow cytometry.

#### 2.4.5 Human MDSCs Preparation and MDSC Suppression Assay

Cord blood was purchased from National Disease Research Interchange (NDRI) and CD34^+^ stem cells were purified with EasySep™ Human cord blood CD34 positive selection kit II (StemCell Technologies). MDSCs were prepared by culturing CD34+ stem cells in RPMI medium supplemented with FLT-3L, GM-CSF, and G-CSF for 9 days. HLA-DR^+^ cells were then depleted from the differentiated cells with an anti-HLA-DR monoclonal Ab per vendor’s instruction (StemCell Tech). The HLA-DR-depleted MDSCs were treated with Imprime (25 μg/ml) or vehicle for 2 hours and then analyzed by flow cytometry and used in the MDSC suppression assay (20). For the MDSC suppression assay, PBMCs prepared from WB were labeled with CFSE, and 2.5 x 10^4^ labeled PBMCs were added to the wells of a 96-well plate and incubated with MDSCs at the indicated PBMC : MDSC ratio in the presence of Dynabeads™ Human T-activator CD3/CD28 (Thermo Fisher Scientific). PBMC proliferation was measured after 3 days by flow cytometry.

### 2.5 Statistics

Statistical analyses for each of the methods are indicated in the respective figure legends. All statistical analyses were calculated using Prism (version 9; GraphPad Software, Inc.).

### 2.6 Study Approval

All mouse protocols were approved by the University of Minnesota Institutional Animal Care and Use Committee (IACUC, Protocol # 2009-38458A). Cord blood samples were purchased from National Disease Research Interchange (Protocol ID # DFRK2 001). Blood samples were obtained from healthy human volunteer subjects with informed consent at Hibercell, Inc. as approved by the New England Institutional Review Board (NEIRB, Study # 120160627).

## 3 Results

### 3.1 Imprime-Induced Production of Cytokines, Chemokines, and Type I IFNs

**Figure 1:**
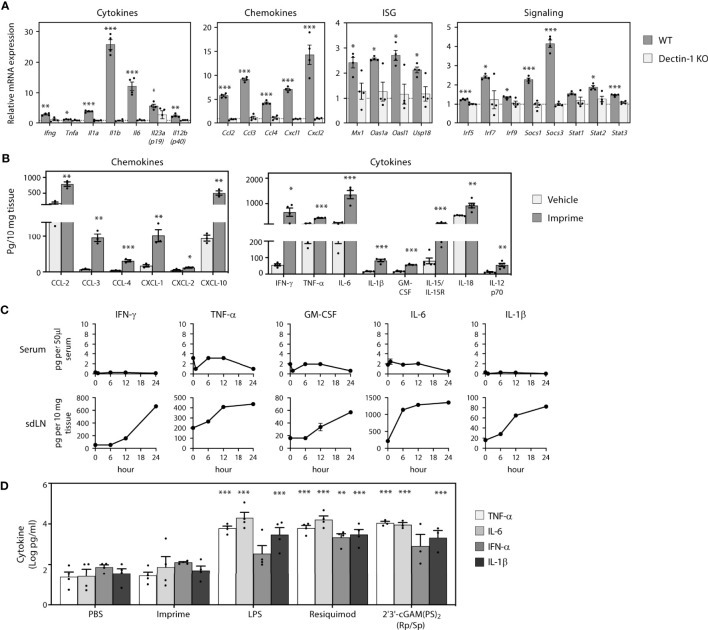
Imprime induces production of cytokines, chemokines, and type I IFNs. Naïve WT or Dectin-1 KO C57BL/6 mice (n = 3-5) were injected i.v. with PBS or 1.2 mg Imprime. **(A)** Transcriptional changes of cytokines, chemokines, ISG and signaling in sdLNs evaluated at 16 hours using a custom QuantiGene Plex and **(B)** chemokine and cytokine proteins were evaluated using the Luminex platform at 24 hours. **(C)** Time course of cytokine production was analyzed in serum and sdLNs. Representative results from ≥ 2 experiments are shown. **(D)** Human whole blood was treated with Imprime, TLR-4 agonist LPS, TLR-7/8 agonist Resiquimod, STING agonist 2’3’-cGAM(PS)_2_ (Rp/Sp) (all at 10 μg/ml), or PBS. Plasma was collected after 24 hours, and cytokines were analyzed by the Luminex platform. Log-transformed cytokine levels for 3 donors were compared. All summary data are presented as mean ± SEM. Multiple *t* tests with Holm-Sidak’s multiple comparison were used for **(A, B)**. Two-way ANOVA with Holm-Sidak’s multiple comparison was used in **(D)**. *p < 0.05, **p < 0.01, ***p < 0.001.

PAMPs are known to induce production of cytokines and chemokines, which is fundamental to inducing a broad inflammatory immune response involving both the innate and adaptive immune systems. As Imprime is a soluble β-glucan PAMP, we first asked what cytokines/chemokines might be induced by Imprime and whether their expression might be influenced by the Dectin-1 receptor, the known receptor for β-1,3-glucans. In order to assess Imprime-induced systemic changes, we administered Imprime i.v. in non-tumor bearing C57BL/6 mice and checked for transcriptional and protein changes in sdLNs and peripheral blood. Sixteen hours post administration of Imprime, increased transcription of numerous chemokines and cytokines, most notably *Ccl2, Ccl3, Ccl4, Cxcl1, Cxcl2*, as well as *Il1b, Il6, and Il23*, was evident in the lymph nodes. Small, but reproducible, increases were evident for *Ifng and Tnfa* (**Figure 1A**). Induction of a Type I IFN gene signature consisting of the interferon stimulated genes *Mx1*, *Oas1a*, *Oasl1* and *Usp18* and increases in the gene of signaling proteins *Irf5, Irf7*, *Irf9*, *Stat1*, *Stat2*, *Stat3*, *Socs1*, and *Socs3* were also consistently evident (**Figure 1A**). These Imprime-induced transcriptional changes were absent in Dectin-1 KO mice (**Figure 1A**). Protein expression for a subset of these chemokines/cytokines was confirmed by ELISA from sdLNs 24 hours post injection (**Figure 1B**). Interestingly, none of the pro-inflammatory cytokines produced in the sdLN were observed in the peripheral blood (**Figure 1C**). This finding led us to further evaluate the cytokine profile of Imprime in human WB to see if Imprime would elicit any of the pro-inflammatory cytokines, and if the profile would be any different in comparison to other PAMPs. Consistent with the *in vivo* findings, Imprime induced a minimal amount of pro-inflammatory cytokines, such as IL-6, TNF-α, and IL-1β, versus those induced by the TLR-7/8 and stimulator of IFN gene agonists (**Figure 1D**). Together, these data demonstrate that Imprime is a unique PAMP in that it does not induce systemic production of significant amounts of the pro-inflammatory cytokines known to be of major safety concern in the clinic.

### 3.2 Imprime Activates NK Cells and Enhances Their Direct Killing Functions

**Figure 2:**
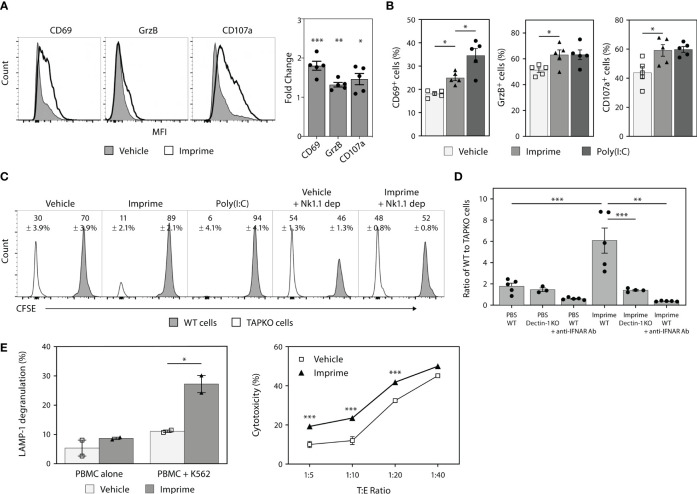
Imprime treatment results in phenotypic and functional activation of mouse and human NK cells. Naïve C57BL/6 mice (n = 5) were injected i.v. with PBS, 1.2 mg Imprime, or 20 µg poly(I:C). **(A)** and **(B)** After 24 hours, expression of activation markers and percent activated cells on NK1.1^+^CD3^-^CD45^+^ splenocytes were analyzed by flow cytometry. **(C)** and **(D)** 24 hours post Imprime or poly(I:C) treatment, mice were injected i.v. with a 1:1 ratio of CFSE-labeled WT to CellTrace™ violet-labeled TAPKO splenic cells, and recipient mice splenic cells were harvested 24 hours post injection. **(C)** Representative histogram and the average percentage change in WT and TAPKO cells with and without NK cell depletion and **(D)** the ratio of WT : TAPKO cells after pre-treatment with an anti-IFNAR Ab or in Dectin-1 KO mice are shown. **(E)** Cytotoxic function of NK cells was measured in human PBMCs using K562 cells as targets. PBMCs pre-treated with Imprime or vehicle for 3 days and then co-incubated with or without K562 cells for 4 hours. Cytotoxicity was measured by flow cytometry using CD107a surface staining (LAMP-1 degranulation) or Live/Dead staining on CD56^+^ NK cells. Representative results from ≥ 2 experiments are shown. All summary data are presented as mean ± SEM. Unpaired Student's t test was used in **(A)** One-way ANOVA with Tukey was used in **(B, D)**, multiple unpaired *t*-test was used in **(E-left)**, Two-way ANOVA with Holm-Sidak’s multiple comparison was used in **(E-right)**. *p < 0.05, **p < 0.01, ***p < 0.001.

Previous *in vivo* tumor studies and *ex vivo* human mechanistic studies have shown that Imprime is capable of enhancing the anti-tumor cytotoxic effector functions of neutrophils and macrophages, including ADCC and Ab-dependent cellular phagocytosis, particularly in the presence of tumor-targeting Abs, such as anti-CD20 Ab (19). As Imprime treatment elicited production of the NK-activating cytokines IL-15/IL-18 (shown in **Figure 1**), we sought to test if Imprime can also activate NK cells. Non-tumor bearing C57BL/6 mice were treated with either PBS (vehicle), Imprime, or Poly(I:C) (positive control). NK cells from the spleens of the mice were analyzed by flow cytometry 24 hours post-treatment. Imprime treatment similarly elicited a significant increase in the MFI and percentage of NK cells expressing the early lymphocyte activation marker CD69, serine protease GrzB, and degranulation marker CD107a (LAMP-1) (**Figures 2A, B**), suggesting that Imprime, like Poly(I:C), can activate NK cell function.

NK cells are very effective at killing MHC class I deficient cells. Accordingly, we assessed NK-mediated killing by mixing splenocytes from WT or MHC class I-deficient TAPKO mice. Each cell type was labeled with different viable cell dyes and adoptively transferred to syngeneic recipient mice in a 1:1 ratio. Twenty-four hours after transfer, the WT : TAPKO cell ratios were 2.3:1 for PBS control-treated mice, indicating the existence of some baseline level of NK activity capable of killing the TAPKO cells (**Figure 2C**). However, Imprime and Poly(I:C) each significantly shifted this ratio in favor of WT cells (~9:1), indicating a higher level of TAPKO killing. Depleting NK cells using the anti-NK1.1 Ab prior to the adoptive transfer of target cells reversed these effects, restoring the WT : TAPKO ratio to ~1:1, thus confirming that the assay was NK cell-dependent. Furthermore, data from Dectin-1 KO and depletion of IFNAR using an anti-IFNAR Ab showed that Imprime-mediated NK-driven killing is Dectin-1- and type 1 IFN-dependent (**Figure 2D**).

We next evaluated the effect of Imprime on human NK cells. As in mice, Imprime treatment of healthy donor PBMCs increased the percentage of LAMP-1^+^ NK cells and also enhanced the killing of K562 cells, the most sensitive and widely used target cells for human NK cell killing, across several different target:effector cell ratios. (**Figure 2E**). Collectively, these results demonstrate that Imprime is capable of phenotypically and functionally activating NK cells.

### 3.3 Imprime Activates Monocytes and Reorients the Immunosuppressive Phenotype and Functionality of Macrophages and Monocyte-Like Myeloid-Derived Suppressor Cells

**Figure 3:**
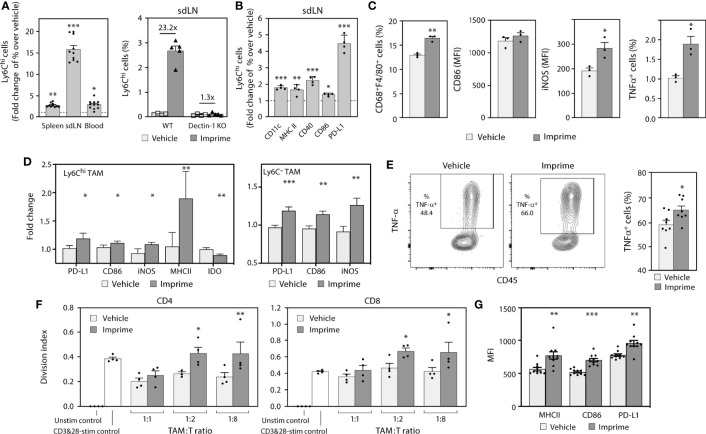
Imprime mobilizes and activates Ly6C^+^ monocytes in tumor-free mice and modulates tumor associated myeloid cells in MC38 syngeneic tumor model. Naïve WT or Dectin-1 KO C57BL/6 mice (n = 3-10) were treated with Imprime or vehicle as described above. **(A)** Fold change over vehicle in frequency of Ly6C^hi^ monocytes in blood, spleens, and sdLNs of WT or Dectin-1 KO mice at 24 hours; **(B)** fold increase in activation markers of Ly6C^hi^ monocytes in sdLN, and **(C)** MFI of activation markers on CD68^+^ F4/80^+^ splenic macrophages at 16 hours and percent of LPS-stimulated TNF-α^+^ expressing splenic macrophages were determined by flow cytometry. **(D)** The MC38 syngeneic mouse model was established as described in Materials and Methods. Imprime or vehicle treatment started when tumors reached ~50mm^3^ (n = 5-8/treatment group). Activation markers on Ly6C^hi^ and Ly6C^-^ TAM analyzed by flow cytometry on day 7 and, **(E)** LPS-induced TNF-α production in TAMs from a representative mouse and bar graph showing data from individual mouse analyzed by intracellular flow cytometry are shown. **(F)** T cell suppression assay was performed as described in Materials and Methods using enriched CD11b^+^ TAM. **(G)** MFI of activation markers on mouse m-MDSC (CD11b^+^Gr-1^lo^Ly6c^+^) were determined by flow cytometry. Representative results from ≥ 2 experiments are shown. All summary data are presented as mean ± SEM. Unpaired Student’s *t* test was used in **(A, B, C, E)**. Multiple *t* tests with Holm-Sidak’s multiple comparison were used in **(D, G)**, and One-way ANOVA was used in **(F)**, and *p < 0.05, **p < 0.01, ***p < 0.001.

In mice, Imprime increased expression of chemokines involved in monocyte function, especially CCL2, which is important for the emigration of Ly6C^hi^ monocytes from the bone marrow into the periphery. We sought to investigate the effect of Imprime on monocyte mobilization, phenotypic activation, trafficking to the secondary lymphoid organs, and the M1/M2 orientation of the macrophages in the spleen of tumor-free mice. Twenty-four hours after i.v. administration in C57BL/6 mice, Imprime was bound to Ly6C^hi^ classical inflammatory monocytes in the spleens and sdLNs of WT mice. As expected, binding was significantly reduced in Dectin-1 KO mice (**Supplementary Figures 2A, B**). We also observed a Dectin-1 dependent increase of frequency of Ly6C^hi^ monocytes in blood, spleen, and sdLNs, with the most profound increase in sdLNs (**Figures 3A**). Furthermore, the Ly6C^hi^ monocytes from Imprime-treated mice showed increased expression of CD11c, CD40, programmed death-ligand 1 (PD-L1), and the classic M1-markers MHC class II and CD86 (**Figure 3B**). Imprime treatment also increased the macrophage numbers in the spleen, and enhanced the expression of M1 markers CD86 and inducible nitric oxide synthase (iNOS). Additionally, these Imprime-educated macrophages were functionally activated, producing more TNF-α in response to LPS stimulation than macrophages from vehicle-treated mice (**Figure 3C**).

We then evaluated the impact of Imprime on monocyte trafficking into a tumor, the phenotype and functionality of macrophages in the tumor immune microenvironment. For this purpose, we used the syngeneic MC38 colon cancer model given its macrophage-rich content and its widespread use to test immuno-oncology therapeutic agents. In MC38-tumor bearing mice, Ly6C^hi^ monocytes were increased in the tumor 3 days after Imprime dosing (**Supplementary Figure 3A**). Tumor gene expression analysis performed 7 days post Imprime dosing showed upregulation of M1 markers, e.g., *Nos2, Il12b*, and *Tnfa*, along with downregulation of M2-associated genes including *Arg1* and *Ccl17* (**Supplementary Figure 3B**). Flow cytometric evaluation of single cell suspensions from tumor tissue showed the presence of macrophages at different stages of differentiation, including the relatively immature (Ly6C^hi^F4/80^+^) macrophages and a smaller percentage of the more mature (Ly6C^-^F4/80^+^) macrophages. Both of these subsets of macrophages showed increased expression of iNOS, CD86, and PD-L1, indicating polarization to an M1-phenotype (**Figure 3D**). Functionally, macrophages from the Imprime-treated mice showed increased TNF-α production following *ex vivo* LPS stimulation (**Figure 3E**). Furthermore, when evaluated in a T cell proliferation assay, the isolated Ly6C^hi^ macrophages were significantly less suppressive to the CD3/CD28-stimulated CD4 and CD8 T cell proliferation (**Figure 3F**). Not surprisingly, the splenic macrophages also showed an M1 phenotype and functionality with increased expression of M1 markers and heightened responsiveness to LPS stimulation (**Supplementary Figure 3C**). Since the Ly6C population encompasses the m-MDSC population, especially in the spleen, we checked for the effect of Imprime on this population by gating out the Ly6C^hi^F4/80^+^ macrophages. Consistent with the effect on macrophages, we found enhanced expression of markers on the m-MDSC population as well, suggesting that Imprime treatment elicits maturation of m-MDSCs to M1 macrophages (**Figure 3G**).

**Figure 4:**
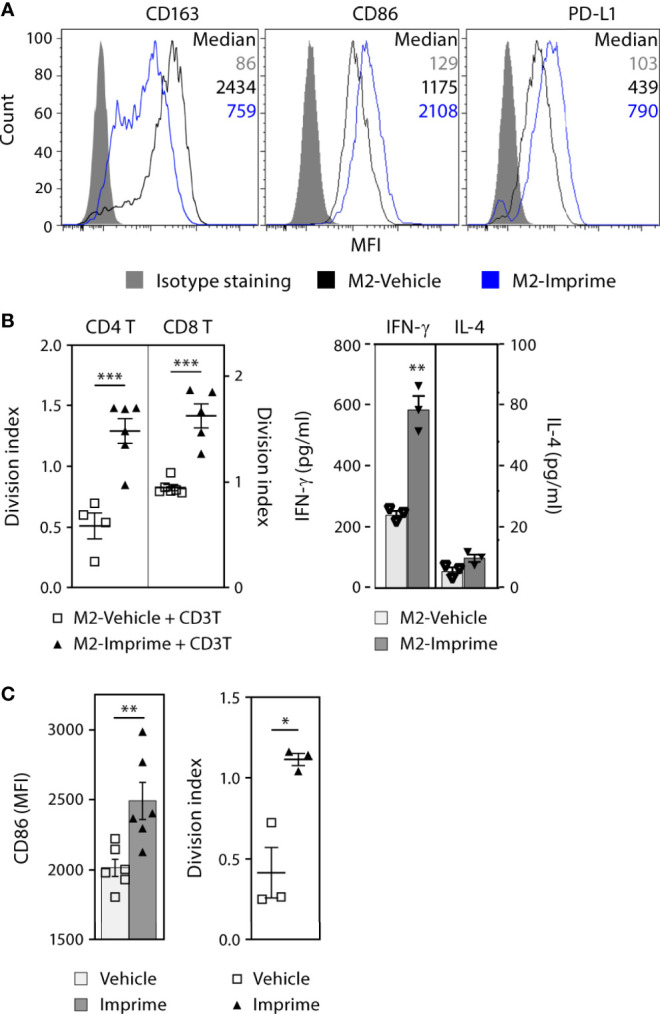
Imprime activates the phenotype and functional state of *ex vivo* human MDMs and MDSCs. MDMs were prepared from monocytes as described in Materials and Methods **(A)** Phenotype of vehicle- or Imprime-treated MDMs was assayed by flow cytometry. **(B)** CD3 & CD28-stimulated CFSE-labeled autologous CD3 T cells were cultured with MDMs for 5 days; T cell proliferation was measured by flow cytometry, and IFN-γ and IL-4 production was analyzed by ELISA. **(C)** MDSCs were prepared from human cord blood as described in Materials and Methods. CD86 expression on MDSCs and functional assessment in a T cell suppression assay were analyzed by flow cytometry. Representative results from ≥ 2 experiments are shown. All summary data are presented as mean ± SEM. Unpaired Student’s *t* test was used. *p < 0.05, **p < 0.01, ***p < 0.001.

The M1-polarization effect of Imprime in mice prompted us to investigate if Imprime has similar effects on human macrophages. The ability of Imprime to bind human monocytes has been shown previously (17). We therefore assessed whether binding of Imprime would influence the phenotype and function of monocyte-derived M2 macrophages. Macrophages derived from Imprime-bound monocytes showed reduced CD163 expression, the classic M2 marker, while increasing expression of the activation (i.e., M1 state) markers CD86 and PD-L1 (**Figure 4A** and **Supplementary Figure 4A**). Accordingly, these Imprime-educated M2 macrophages stimulated expansion of CD4 and CD8 T cells and also increased expression Th1 cytokine IFN-γ with no increase in the Th2 cytokine IL-4 (**Figures 4B**). We next tested whether Imprime could foster this repolarization and drive T cell expansion in the presence of immunosuppressive cytokine/growth factor-containing TCM harvested from the culture of the human pancreatic cancer cell line BxPC-3, a cell line demonstrated to produce immunosuppressive cytokines such as VEGF and as such induce M2 polarization (**Supplementary Figure 4B** and (21–23)). Indeed, Imprime treatment still elicited M1 polarization as well as T cell expansion (**Supplementary Figure 4C**). Increased CD86 expression were also observed with the TCM from another pancreatic cancer cell line MiaPaCa (data not shown). We extended these findings to the impact of Imprime on MDSC function using human cord blood-derived MDSCs. As with the *ex vivo* treatment of healthy human MDM, Imprime treatment of these cord blood MDSCs enhanced expression of CD86 while re-programming the function of these Imprime-educated MDSCs, alleviating MDSC-mediated suppression of T cell expansion (**Figures 4C**).

Taken together, these results show that Imprime shifts the macrophages and MDSCs toward a more M1-like phenotype and consequently, more permissive to anti-tumor T cell immunity.

### 3.4 Imprime Binds to and Facilitates Maturation of Primary DCs and MoDCs

**Figure 5:**
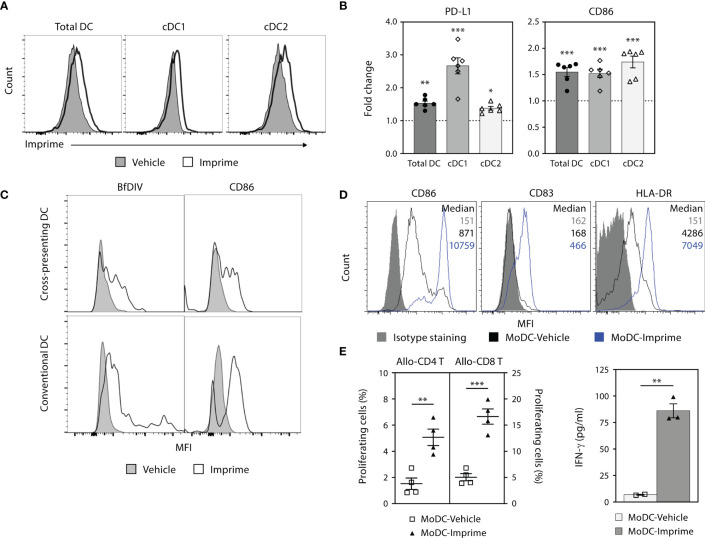
Imprime binds and activates mouse and human DC. **(A)** and **(B)** C57BL/6 mice (n = 3-5) were treated with Imprime or vehicle as described above. 24 hours post administration, Imprime binding of mouse total DC, cDC1, and cDC2, as well as expression of activation markers PD-L1 and CD86 were analyzed by flow cytometry. **(C)** Imprime binding in a human subject was detected with an anti-glucan Ab BfDIV and analyzed by flow cytometry. MoDCs were prepared from human WB and vehicle- or Imprime-treated MoDCs were subsequently evaluated for **(D)** phenotype, and **(E)** functions including allogeneic mixed lymphocyte reaction, and modulation of IFN-γ production. Representative results from ≥ 2 experiments are shown. All summary data are presented as mean ± SEM. Multiple *t* tests with Holm-Sidak’s multiple comparison was used in **(B)**, and Unpaired Student’s *t* test was used in **(E)**. *p < 0.05, **p < 0.01, ***p < 0.001.

Upon engagement with PAMPs, professional antigen presenting cells (APCs) like DCs mature and upregulate co-stimulatory markers (e.g., CD80/86 and CD40) that are central to T cell activation and differentiation. We therefore evaluated the ability of Imprime to bind and activate DCs, in both murine and human. In C57BL/6 mice, Imprime binding was readily apparent 2 hours post-dosing on conventional DCs, conventional type 2 DCs (cDC2) and cross-presenting conventional type 1 DCs (cDC1) in sdLNs (**Figure 5A** and **Supplementary Figure 5A**). These DCs also exhibited increased expression of the activation/maturation markers CD86 and PD-L1 (**Figure 5B**).

Imprime binding to the human counterpart DC populations was tested in human WB. Imprime bound both the cross-presenting (CD141^+^CD11c^+^) and conventional DC (CD1c^+^CD11c^+^) and increased CD86 expression with a degree of variability (**Figure 5C** and **Supplementary Figure 5A**). Similarly, Imprime also elicited maturation on MoDCs, upregulating maturation and activation markers including CD83, HLA-DR, and CD86 (**Figure 5D** and **Supplementary 5B**). Furthermore, Imprime treatment significantly enhanced the ability of these MoDCs to drive T cell proliferation and IFN-γ production in an allogeneic mixed lymphocyte reaction (**Figures 5E**). We also found that Imprime-bound DC had elevated levels of activation markers even in the presence of BxPC-3-TCM demonstrating that Imprime could potentially be able to counter VEGF’s ability to hamper differentiation of DCs in the tumor microenvironment (**Supplementary Figure 5C**).

Collectively, these results show that Imprime treatment can facilitate the maturation of DCs, including both cDC1 and cDC2 lineages, which are particularly important for the activation of CD8 and CD4 T cells, respectively.

### 3.5 Imprime Drives Antigen-Specific T Cell Responses

Because the different causes of Imprime-mediated immune activation are fundamental to priming a *de novo* T cell response as well as enhancing existing T cell immunity, we next investigated whether Imprime treatment could activate CD8 T cell responses using the well-studied chicken OVA antigen model antigen as well as the MC38 tumor model.

**Figure 6:**
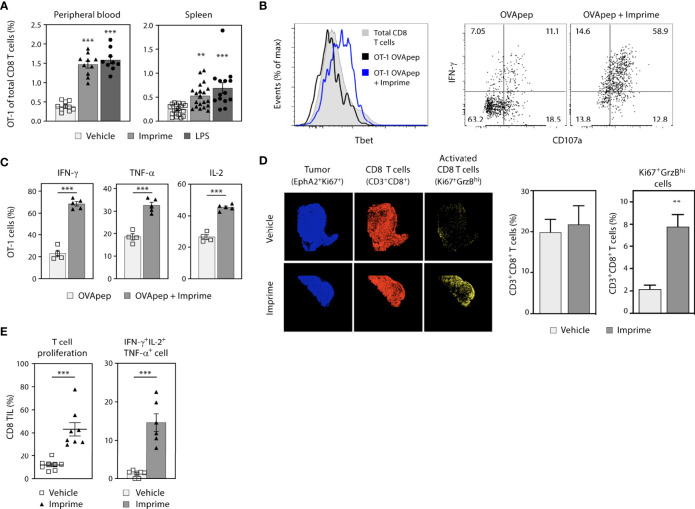
Imprime drives antigen-specific CD8 T cell priming and enhanced effector T cell responses in the tumor. Naïve phenotype CD44^lo^ OT-1 CD8 T cells were adoptively transferred into congenic WT or Dectin-1 KO recipient mice (n = 4-10). Mice were then immunized i.v. with OVA_257-264_/H-2K^b^ peptide and either vehicle, Imprime, or LPS. **(A)** Percent OT-1 cells of total CD8 T cells in peripheral blood (day 5) and spleen (day 7), **(B)** Tbet expression, IFN-γ production and CD107a expression in splenocytes, and **(C)** percent splenic OT-1 making IFN-γ, TNF-α, or IL-2 after *in vitro* OVA peptide stimulation for 5 hours determined by flow cytometry are shown. **(D)** Immunohistochemistry showing tumor cells (blue), CD8^+^ T cells (red), and activated CD8^+^ T cells (yellow), and the quantitative analysis of infiltrated T cells in MC38 tumor-bearing mice treated with Imprime or vehicle, are shown. **(E)** Proliferation of CD3/CD28-stimulated tumor infiltrating lymphocytes (TIL) as determined by CellTrace™ violet dilution, and cytokine production of CD8+ T cells analyzed by intracellular flow cytometry are shown. Representative results from ≥ 2 experiments are shown. All summary data are presented as mean ± SEM. One-way ANOVA and Dunnett multiple comparison was used in **(A)**, Unpaired Student’s *t* test was used in **(C, E)**. **p < 0.01, ***p < 0.001.

Congenically-marked OT-1 CD8 T cells specific to OVA_257-264_/H-2K^b^ peptide (OVApep) were adoptively transferred into syngeneic recipient C57BL/6 mice that were then immunized i.v. with this OVApep +/- Imprime or the positive control TLR-4 agonist LPS. When co-injected with OVApep, both Imprime and LPS promoted expansion of OT-1 CD8 T cells, which coincided with upregulation of the transcription factor Tbet (**Figures 6A, B**). Following *in vitro* peptide stimulation 7 days after immunization, OT-1 primed in the presence of Imprime showed increased degranulation *via* CD107a expression and produced more IFN-γ, TNF-α, and IL-2 (**Figures 6C**). Additionally, the ability of Imprime to promote OT-1 cell expansion and effector function was dependent on Dectin-1 (**Supplementary Figure 6**). These data indicate that Imprime treatment can drive antigen-specific expansion and differentiation of CD8 T cells.

We next assessed the ability of Imprime to activate CD8 T cells in the MC38 syngeneic tumor model by tracking expression of both GrzB and Ki67 in tumor tissue using multi-channel immunofluorescence. In mice treated with Imprime, a significantly higher percentage of CD8 T cells expressed both Ki67 and GrzB (**Figure 6D**), and when isolated and stimulated with anti-CD3/CD28 monoclonal Abs, exhibited enhanced proliferation and production of critical anti-tumor effector cytokines IFN-γ, IL-2, and TNF-α (**Figure 6E**).

These data collectively demonstrate that Imprime treatment can elicit a robust T cell immune response marked by antigen specificity and heightened effector functions.

### 3.6 Imprime Enhances the Therapeutic Efficacy of Anti-Tumor, Anti-Angiogenic, and ICI Monoclonal Abs

**Figure 7:**
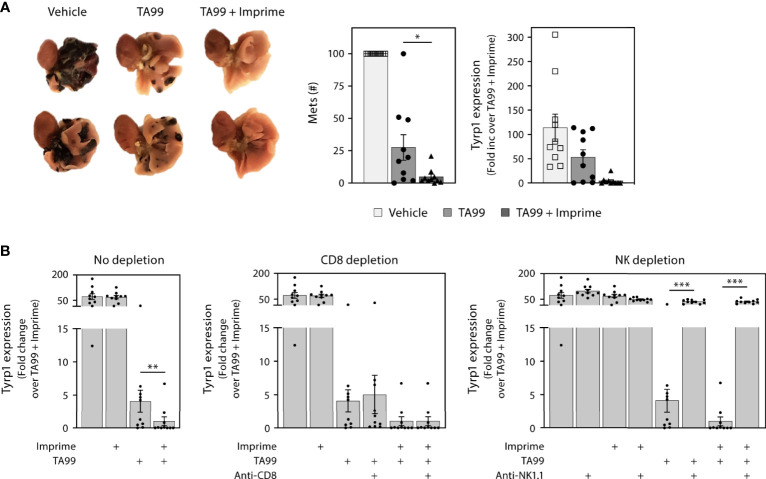
Imprime in combination with a tumor-targeting mAb inhibits formation of melanoma metastases. One x 10^5^ B16F10 cells were administered i.v. to naïve C57BL/6 mice (n = 10 per treatment group), and treatments were administered as described in Materials and Methods. Mice were euthanized on day 21 and lungs were removed. **(A)** Representative picture of day 21 mouse lungs from different treatment groups; two samples from each treatment group are shown. The number of metastases (mets) in the lungs were counted or evaluated by quantitative RT-PCR using Tyrp1 expression. **(B)** Relative expression of Tyrp1 message in the day 21 lungs, upon CD8 T cell depletion or NK depletion. Representative results from 2 experiments are shown. All summary data are presented as mean ± SEM. One-way ANOVA and Tukey multiple comparison was used in **(A, B)**. *p < 0.05, **p < 0.01, ***p < 0.001.

To explore the capability of Imprime treatment to enhance the efficacy of tumor-targeting Ab therapy, we first chose to combine Imprime treatment with the specific tumor-targeting Ab TA99 in the well-studied B16F10 melanoma (B16) experimental metastasis model. In this model, the therapeutic efficacy of the TA99 Ab, which specifically recognizes the Tyrp1 antigen expressed by B16 melanoma cells, has been shown to be dependent upon NK activity and ADCC (17). While TA99 alone resulted in a significant, but incomplete decrease in the number of nodules of lung metastases as measured by visual counts or Tyrp1 melanoma antigen measured by RT-PCR, Imprime alone did not cause a decrease in lung metastases or Tyrp1 expression. But, when combined with TA99, Imprime combination therapy nearly eliminated the lung metastases and Tyrp1 expression (**Figures 7A**). Depletion of NK cells with the anti-NK1.1 Ab obviated efficacy in mice treated with TA99 or the Imprime + TA99 combination (**Figure 7B**). CD8 T cell depletion did not influence the efficacy of TA99 alone or the TA99 + Imprime combination indicating that efficacy in this model is driven by NK cell activity as previously published (24). These data provide evidence that Imprime can enhance the therapeutic efficacy of tumor-targeting Abs that are designed to leverage the killing functions of the innate immune system, specifically NK cells.

**Figure 8:**
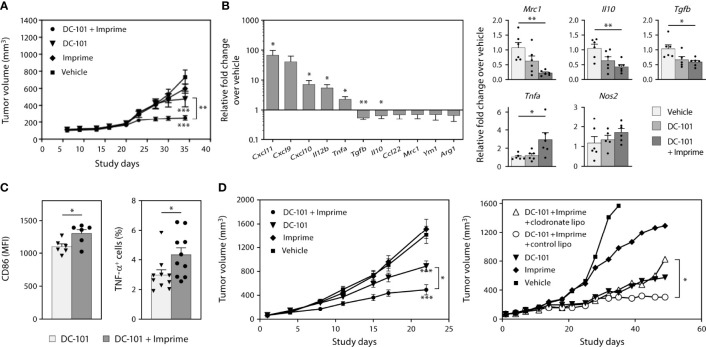
Imprime remodulates tumor microenvironment and influences anti-tumor efficacy of anti-angiogenic agent in xenograft models. As described in Materials and Methods, H441 and H1299 non-small cell lung cancer xenograft tumor bearing nude mice were treated with anti-VEGF receptor 2 Ab DC-101 and/or Imprime (n = 10). **(A)** Tumor growth in the different treatment groups of the H441 model. **(B)** Changes in mRNA expression of M1/M2 markers of Imprime versus vehicle-treated tumor cell suspension, and changes in mRNA expression of M1/M2 markers of DC-101 versus DC-101+ Imprime and vehicle in tumor were evaluated. **(C)** Functional activities of TAMs were measured by CD86 expression and percent TNF-α^+^ of LPS-stimulated TAMs using flow cytometry. **(D)** Tumor growth in the different treatment groups of the H1299 model and the effect of macrophage depletion on tumor growth using clodronate liposomes or control liposomes (n ≥ 7) were evaluated. Representative results from 2 experiments are shown. All summary data are presented as mean ± SEM. Two-way ANOVA analysis and Tukey multiple comparison were used in **(A, D)**, Unpaired Student’s *t* test was used in **(B-left, C)** and One-way ANOVA with Dunnett multi-comparation was used in **(B-right)**. *p < 0.05, **p < 0.01, ***p < 0.001.

We next evaluated whether Imprime may enhance the therapeutic activity of anti-angiogenic therapy targeting VEGF. Previous work in human cancer xenograft models had suggested Imprime could enhance the efficacy of the anti-VEGF Ab bevacizumab (25). For these studies, we sought to extend and advance those findings to provide additional mechanistic insight using the anti-VEGFR2 Ab DC-101. As agents targeting the VEGF-VEGFR axis have been shown to modulate immunosuppressive myeloid cells (4), we reasoned that the combination of anti-VEGF therapy with Imprime may not only have enhanced anti-tumor effects but also have a more profound impact on the immunosuppressive, wound-healing tumor microenvironment than either agent alone. We first evaluated the combined efficacy of Imprime and DC-101 in two NSCLC xenograft models, H441 and H1299. The xenograft models provided the opportunity to isolate the impact of this combined therapy on myeloid cells. In the H441 model, suppression of tumor growth was particularly pronounced in the combination treatment group (**Figure 8A**). We then checked whether Imprime treatment alone would impart M1 characteristics to the tumor, and as shown in **Figures 8B**, Imprime treatment versus vehicle treatment upregulated several M1-related genes, such as *Il12b, Tnfa*, and *Cxcl9/10/11*, and downmodulated M2 genes, including *Tgfb, Il10, Ccl22, Mrc1, Ym1, and Arg1*. We confirmed the modulation of selected genes in the Imprime + DC-101 combination treatment group as shown by increased expression of M1 genes *Nos2 and Tnfa* and downmodulation of M2 genes *Mrc1, Il10, and Tgfb.* The M1 phenotype and functionality of tumor infiltrating macrophages were further confirmed by flow cytometric detection of high CD86 expression and increased TNF-α production post-LPS stimulation (**Figures 8C**). In the second xenograft model, H1299, we checked for the anti-tumor efficacy of Imprime and DC-101 after confirming the upregulation of M1-related genes with Imprime treatment (data not shown). As shown in **Figure 8D**, the Imprime + DC-101 treatment group showed significant tumor growth inhibition, and this combined efficacy was abrogated in the mice treated with clodronate liposomes to deplete the phagocytic cells, further highlighting the critical role of myeloid cells in Imprime’s anti-tumor mechanism of action. These data show that Imprime-based treatment prompts a shift in the immune microenvironment of a tumor *in situ*, eliciting enhanced tumor growth inhibition in concert with anti-angiogenic therapy.

**Figure 9:**
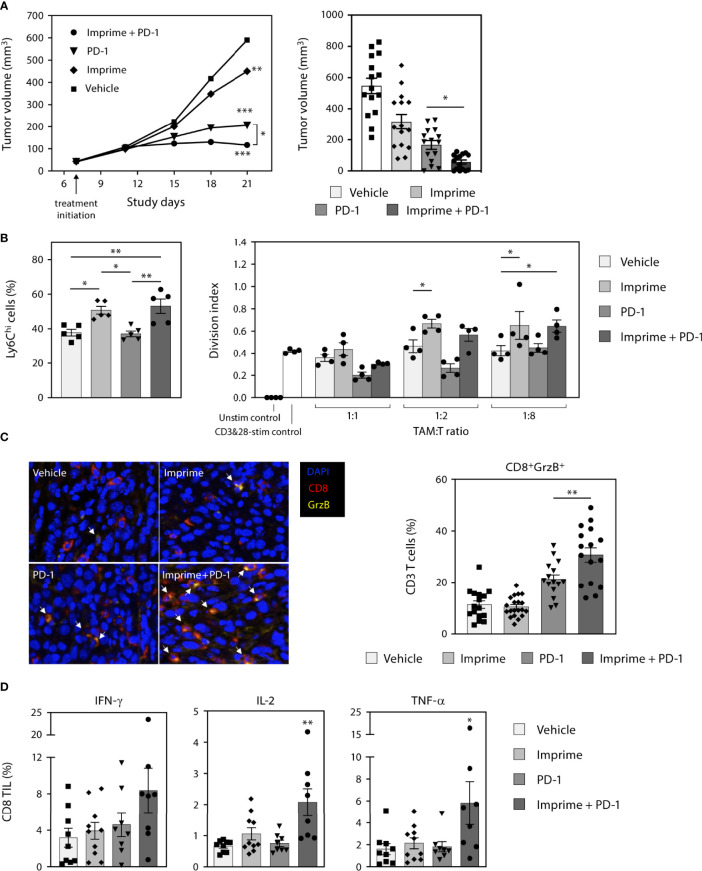
Imprime synergizes with anti-PD-1 Ab therapy in the murine MC38 tumor model. **(A)** Tumor growth curve in the different treatment groups (n ≥ 10) in the MC38 tumor model is shown on the left. Data is representative of 3 separate experiments. Tumor volume at day 21 are shown in the bar graph on the right. Each symbol in the graph represents an individual mouse. **(B)** Analysis of the Ly6C^hi^ myeloid population present in the tumor, and functional analysis of TAMs in a T cell suppression assay are shown. **(C)** Immunohistochemistry data for infiltrating activated CD8 T cells are shown as an image and quantitative data presented in the graphical form. Data for each mouse were calculated using 50% coverage of the tumor. **(D)** Percent CD8 tumor infiltrating lymphocytes (TIL) from each treatment group were evaluated for the expression of IFN-γ, IL-2, and TNF-α after stimulation with MC38 peptide for 5 hours. For **(B-D),** representative results from 2 experiments are shown. All summary data are presented as mean ± SEM. Two-way ANOVA using Holm-Sidak for multiple comparison was used in **(A-left)**, One-way ANOVA using Holm-Sidak for multiple comparison was used in **(A-right)**, One-way ANOVA using Tukey for multiple comparison was used in **(B-left, C, D)**, Two-way ANOVA with Dunnett for multiple comparison was used in **(B-right)**. *p < 0.05, **p < 0.01, ***p < 0.001.

Our findings in this study consistently demonstrated that Imprime upregulated the compensatory immune checkpoints such as PD-L1 on both the tumor and myeloid cells. We reasoned that Imprime’s mechanism is well positioned to synergize with ICI therapy. To test this hypothesis, we examined the combination of Imprime with the ICI anti-PD-1 in the MC38 model. As previously reported, anti-PD-1 alone caused significant tumor reduction while Imprime alone only had a modest effect relative to vehicle. When combined, we found Imprime synergized with anti-PD-1 therapy to further reduce tumor growth (**Figure 9A**). Cumulative results showed that increased number of mice had cleared tumors after 2 weeks of treatment in the Imprime + anti-PD1 treatment group and upon rechallenge with MC38 tumor cells in the opposite flank, only naïve controls developed palpable tumors, demonstrating that more mice in the combination group had developed memory response (**Supplementary Figure 7**). In both the Imprime and Imprime + PD-1 groups, there was a significant increase in the percentage of Ly6C^hi^ monocytes within these tumors compared to either vehicle or anti-PD-1 group. Importantly, the isolated TAMs (CD11b^+^F4/80^+^) from both Imprime and Imprime + PD-1-treated groups were significantly less suppressive than those from the vehicle and anti-PD-1 alone group (**Figure 9B**). Tumor imaging by immunohistochemistry showed an increase in the percentage of activated CD8 T cells (GrzB^+^) was achieved in the combination group (**Figure 9C**). Furthermore, the infiltrating CD8 T cells in the Imprime + anti-PD-1 combination group showed the highest production of effector cytokines IFN-γ, TNF-α, and IL-2 (**Figure 9D**) when stimulated by MC38-specific antigen peptide. These results provided further evidence of Imprime’s ability to induce both the recruitment and polarization of myeloid cells to create and sustain an immune microenvironment supportive of antigen-specific T cell immune response.

## 4 Discussion

In the current T cell-centric landscape of immunotherapy, the role of innate immune modulators is being increasingly appreciated as a clinically viable and relevant therapeutic class of therapies. Rather than targeting a singular mechanistic defect in the immune response, stimulating the innate immune system can trigger the natural multi-tiered immune response that is required to mount its own anti-tumor effector responses, and also for the onset and sustenance of T cell immunity (26, 27). Innate immunity invokes a multitude of effector functions such as phagocytosis, ADCC, and antigen presentation to T cells by various types of myeloid and lymphoid lineage cells, including neutrophils, NK cells, monocytes/macrophages, and DCs. Pattern recognition receptor (PRR)-PAMP mediated inflammatory signaling in these innate immune cells is fundamental to instigating the direct anti-tumor effector functions and bridging to adaptive immunity by supporting the development of deeper, long-lasting T cell immunity (28). Natural exogenous or synthetic PAMP agonists are currently in clinical development, mainly as agents that are delivered intratumorally and in combination with agents such as T cell-targeting immunomodulators. In this study, we have presented pre-clinical mechanistic data in support of, and aligned with, our clinical rationale. These data illustrate that Imprime, a systemically delivered, natural yeast-derived β-glucan PAMP, can be used in combination with a number of combinatorial agents; tumor-targeting Abs, anti-angiogenics, and ICIs.

Imprime is unique among PAMPs in that it is i.v. administered and thus provides systemic activation of the innate immune system. As expected, *in vivo* dosing of Imprime induced the hallmarks of innate immunity, which included the production of pro-inflammatory cytokines and chemokines required for trafficking of myeloid cells, and type I IFN signature in the lymph nodes. However, in comparison to the other PAMPs, Imprime did not elicit appreciable levels of the pro-inflammatory cytokines, such as IL-6, IL-1β, and TNF-α, in the peripheral blood. It is plausible that the localized production of pro-inflammatory cytokines in the lymph nodes is due to significant trafficking of inflammatory Ly6C^hi^ monocytes to lymph nodes *vs* spleen and peripheral blood. This is a critically important feature of Imprime, given that the other PAMPs, including TLR agonists, are delivered as intratumoral agents because of their potential to elicit dangerous “cytokine storms” when delivered i.v (6–8). Additionally, intratumoral administration may limit these drugs to tumors that are accessible by injection and requires abscopal effects for targeting distant metastases. On the contrary, Imprime has been administered to healthy volunteers safely and also in late-stage cancer patients with widespread visceral metastases. Peripheral blood cytokine profiling in these healthy volunteers and cancer patients has shown consistent chemokine-centric and less of a pro-inflammatory cytokine profile (16, 29, 30). In healthy volunteers, the most common adverse events experienced following Imprime dosing were mild to moderate infusion reactions that were manageable and reversible (15, 16). In clinical studies of differing tumor types and therapy combinations (tumor-targeting Abs, anti-angiogenic agents, checkpoint inhibitors or chemotherapies), the reported serious adverse events were qualitatively consistent with those observed with the combination drugs (10, 12, 13, 15, 16).

The Imprime-induced type I IFN signature is consistent with previously published studies that have shown a role for type I IFN in Dectin-1-mediated protection against fungal pathogens and specifically, the regulation of CD8 T cell-based immunity that is triggered by fungal β-glucan particles (31–35). β-Glucan-induced type I IFN production, specifically IFN-β, has been shown to activate both monocytes and DCs (31, 34). Imprime treatment also mobilized and activated Ly6C^hi^ inflammatory monocytes in peripheral blood and secondary lymphoid organs in tumor-free mice within 24 hours of treatment. Ly6C^hi^ monocytes from Imprime-treated mice showed upregulation of markers associated with activation and polarization to the pro-inflammatory M1 phenotype (i.e., MHCII, CD40, and CD86), as did splenic macrophages (i.e., increased iNOS and TNF-α). True to the attribute of a PAMP, Imprime administration also activated DCs with increased expression of MHCII and CD86 in response to Imprime, indicating a mature APC phenotype. Finally, in the OT-1 model, Imprime was also able to mediate antigen-specific T cell priming in a Dectin-1 dependent manner. Together, these data indicate that Imprime binds to and modulates a wide array of innate subsets, especially APCs, resulting in the functional priming of CD8 T cells. Not surprisingly, Imprime upregulated PD-L1 expression on both the monocyte and DC subsets; it is conceivable that this increased expression is downstream of Imprime-induced type I IFN induction. Given the role of IFN-α and IFN-β in the regulation of PD-L1 expression, further mechanistic studies are warranted to understand the differential role of IFN-α versus IFN-β in Imprime’s mechanism (36, 37).

While the Dectin-1 receptor is predominantly expressed on monocytes, macrophages, and DCs, NK cells have been shown to have low levels of Dectin-1 (38). However, Dectin-1 signaling in DCs was demonstrated to activate cytotoxic function in NK cells (39). Given that Imprime administration resulted in production of IFN-γ and other key regulators of NK maturation and activity, IL-15 and IL-18, it is plausible that Imprime-induced myeloid expression of IL-15/IL-18 led to increased CD69, GrzB, and CD107a (LAMP-1), which are hallmarks of mature and functional cytotoxic NK cells. The finding of NK cell-mediated killing of TAP-deficient cells after Imprime treatment being Dectin-1 and type I IFN response-dependent suggested that the effect was dependent on myeloid cells. Furthermore, Imprime’s activating effect on human NK cells observed only in PBMCs versus isolated NK cells (data not shown) further lend credence to the potential of Imprime indirectly activating NK cells *via* myeloid cells.

Consistent to the observations in non-tumor bearing mice, the phenotypic and functional features stimulated by Imprime were also observed in MC38 tumor-bearing mice. Imprime treatment in the MC38 tumor model resulted in increased frequency of Ly6C^hi^ monocytes and also upregulation of M1 markers on the spectrum of immature and mature macrophages as evaluated by gene expression analyses, phenotypic changes, and functional activity. TAMs isolated from Imprime-treated MC38 tumors not only displayed phenotypic activation but were also less inhibitory to T cell proliferation compared with vehicle-treated tumors. These repolarization effects of Imprime were also observed systemically on splenic macrophages, and immature myeloid cells also exhibited the M1 phenotype post Imprime treatment. As most macrophages in the tumor microenvironment are Ly6C^hi^, it can be inferred that these are recruited macrophages, but further studies using lineage tracing and adoptive transfer of Ly6C^hi^ monocytes are needed to confirm these findings. Imprime’s establishment of this immuno-supportive microenvironment also allowed increased trafficking of activated cytotoxic T cells (CD8^+^/Ki67^+^/GrzB^+^) with effector phenotype (IFN-γ^+^/IL-2^+^/TNF-α^+^) into the tumor.

The aforementioned phenotypic and functional effects elicited by Imprime make it an attractive candidate to combine with therapeutics from other mechanistic classes. Here we have shown in four different preclinical tumor models that Imprime is capable of synergizing with multiple different classes of therapeutic agents. In the B16 melanoma model, which has been characterized to be MHC class I-defective and highly refractory to ICIs (40), Imprime combined with the anti-Tyrp1 Ab TA99 almost completely prevented outgrowth of lung metastases. In this model, the role of NK cells was crucial and likely was mediated by ADCC as the TA99 Ab was also required for significant efficacy to be achieved. In two separate xenograft models, H441 and H1299, Imprime showed enhanced efficacy with the anti-VEGFR2 Ab DC-101. Anti-angiogenic Abs, such as the anti-VEGF bevacizumab and anti-VEGFR2 ramucirumab, have been shown to block VEGF signaling, “normalizing” the tortured, disorganized vasculature typical of advanced cancers, and helping shift the immune microenvironment from one that is immunosuppressive to one that promotes immune function, tumor recognition, and DC maturation (41). We showed that Imprime, by activating innate immune cells, may work in concert with anti-angiogenics to repolarize the immunosuppressive microenvironment and to enable a more robust anti-cancer immune response. Finally, in the syngeneic MC38 colorectal model, Imprime not only showed superior reduction of tumor growth when combined with an anti-PD-1 Ab but immune correlative assessment demonstrated that TAMs isolated from Imprime + anti-PD-1 combination-treated tumors were also consistently less suppressive towards CD8, enabling them to take on a more effector phenotype (IFN-γ^+^IL-2^+^TNF-α^+^) when presented with MC38-specific antigens. Further work of depleting specific innate immune cell subsets is warranted to delineate the role of monocytes/macrophages versus DC subsets in the enhanced combinatorial efficacy of Imprime and anti-PD-1 Ab. Consistent with the mouse studies, *ex vivo* human WB studies further confirmed Imprime’s ability to elicit a robust response in multiple different innate immune cells types to establish a “primed” immune state; enhanced NK cytotoxic functions, functionally repolarize macrophages from M2 to more M1-like, enhance maturation of both primary DC and MoDCs, and even promote human cord-blood derived MDSC to a more APC-like phenotype, all of which then exhibited less inhibitory properties to CD8 T cell functions.

It is important to note that the combinatorial benefit observed in pre-clinical tumor models with Imprime and tumor targeting antibodies, anti-angiogenics, and immune checkpoint inhibitors has translated into the clinic where early signals of efficacy have been observed in cancer patients following treatment with respective combinations. In high-risk chronic lymphocytic leukemia patients (including those with del 17p, del 11q risk factors), the addition of Imprime to the tumor-targeting Abs rituximab and alemtuzumab yielded a complete response rate of 65% compared to a 36% complete response observed with rituximab and alemtuzumab in a similar trial run at the same institution during the same time period (10, 11). First-line advanced nonsquamous non-small cell lung cancer (NSCLC) patients treated with Imprime in combination with the anti-angiogenic agent bevacizumab and chemotherapy, including carboplatin and paclitaxel, yielded an increased overall response rate and improved median overall survival compared to the respective endpoints of the control group treated with bevacizumab, carboplatin, and paclitaxel (12). Lastly, the primary analyses results of a recently completed single arm trial, IMPRIME-1 (NCT02981303) showed that Imprime in combination with an ICI, the anti-programmed cell death protein (PD-1) Ab pembrolizumab in metastatic triple negative breast cancer (TNBC) patients yielded an enhanced 12-month overall survival rate (57.6%) and increased median overall survival (16.4 months). While pembrolizumab monotherapy results of 39.8% 12-month overall survival rate and 9.0 month median overall survival were reported in Keynote-86 phase 2 study (13, 14). Mechanistic proof-of-concept data for M1 polarization and increased infiltration of activated T cells post treatment with the Imprime/pembrolizumab combination was obtained by multispectral immunofluorescence analyses of the paired baseline and 6-week post-treatment biopsy samples (AACR 2020 abstract) (13). Imprime in combination with pembrolizumab is currently being evaluated in a Phase 2 trial in HR+/Her2- metastatic breast cancer patients who have progressed through prior hormonal therapy (NCT05159778). HR+/Her2- breast tumors are considered to have an “immunologically cold” microenvironment, which may predict low responsiveness to monotherapy with immune checkpoint inhibitors and the clinical hypothesis in the trial is that just like the observations in the pre-clinical models and TNBC patients in the clinic, the combination of Imprime/pembrolizumab will be able to reprogram the immunosuppressive microenvironment and allow increased trafficking of activated T cells in these patients as well.

Overall, we have provided evidence that Imprime is a PAMP and broadly activates multiple innate immune cells and functionalizes them towards an anti-tumor response. One of the gaps in this study is that we did not deplete specific immune subsets to understand their contribution to the anti-tumor activity with each of the combination. Additionally, the study also lacks the understanding of the molecular mechanism of activation in the different myeloid lineage cells. Particulate β-glucans have been demonstrated to induce Dectin-1-mediated trained immunity, a “non-tolerizing” activation of innate immune cells by epigenetic modifications and metabolic reprogramming (42, 43). More recently, trained immunity has been shown to be instrumental in multiple cancer therapies, including BCG, and ICIs (44, 45). Ongoing mechanistic work on the ability of Imprime, a soluble β-glucan to instill trained immunity in multiple lineages of innate immune cells will further shed light on the broad applicability of Imprime with several combinatorial agents in several different tumor histologies.

## Data Availability Statement

The original contributions presented in the study are included in the article/**Supplementary Materials**. Further inquiries can be directed to the corresponding author.

## Ethics Statement

The studies involving human participants were reviewed and approved by The New England Institutional Review Board. The patients/participants provided their written informed consent to participate in this study. The animal study was reviewed and approved by The University of Minnesota Institutional Animal Care and Use Committee.

## Author Contributions

NB, RF, MU, JG, AC, and KG contributed to the conception and design of the study. AC, XQ, RF, KG, YY, and TK developed the methodology. TK, AC, XQ, NO, RF, and YY acquired the data. XQ, NO, RF, MD, AC, KG, YY, and NB analyzed and interpreted data. NB, AC, MU, YY, and RF wrote, reviewed, and/or revised the manuscript. MD, TJ, and KM provided administrative, technical, or material support. NB, MU, and JG supervised the study. All authors contributed to the article and approved the submitted version.

## Funding

This study received funding from Biothera Pharmaceuticals, Inc. and HiberCell, Inc. Biothera Pharmaceuticals, Inc provided funding for all the experiments. HiberCell, Inc. funded the writing of this manuscript.

## Conflict of Interest

All authors were employed by Biothera Pharmaceuticals, Inc.. Authors AC, TK, XQ, MD, TJ, KM, NB are currently employed by HiberCell, Inc.

## Publisher’s Note

All claims expressed in this article are solely those of the authors and do not necessarily represent those of their affiliated organizations, or those of the publisher, the editors and the reviewers. Any product that may be evaluated in this article, or claim that may be made by its manufacturer, is not guaranteed or endorsed by the publisher.
