# Supplementary material for: Imprime PGG Enhances Anti-Tumor Effects of Tumor-Targeting, Anti-Angiogenic, and Immune Checkpoint Inhibitor Antibodies
Source: Front Oncol. 2022 May 26;12:869078. doi: 10.3389/fonc.2022.869078 (PMC9178990; doi:10.3389/fonc.2022.869078)
Supplement: Supplementary file 1 [file DataSheet_1.pdf]

Supplemental Table 1. List of antibodies used in the study

Fluorochrome	Specificity	clone	vendor	Cat #
Brilliant Violet 510	Mouse CD4	GK1.5	Biolegend	100449
PE/Cy5	Mouse CD8a	53-6.7	Biolegend	100710
FITC	Mouse CD11c	REA754	Miltenyi Biotec	130-110-700
Purified	Mouse CD16/CD32 (Fc Shield)	2.4G2	Tonbo Biosciences	70-0161-U500
PE/Cy7	Mouse CD24	M1/69	Biolegend	101821
Alexa Fluor 647	Mouse CD40	3/23	Biolegend	124614
APC/Cy7	Mouse/ human CD44	IM7	Biolegend	103028
Violet Fluor 450	Mouse/ human CD44	IM7	Tonbo Biosciences	75-0441-U100
FITC	Mouse CD45	I3/2.3	Biolegend	147710
PerCP-Cyanine 5.5	Mouse CD45.1	A20	Tonbo Biosciences	65-04530-U100
PE-Cy5	Mouse CD69	H1.2F3	Biolegend	104509
Brilliant Violet 785/ PE/Cy5	Mouse CD86	GL-1	Biolegend	105043/ 105016
APC/Cy7	Rat CD90/ mouse CD90.1 (Thy-1.1)	OX-7	Biolegend	202520
Alexa Fluor 700	Mouse CD90.2	30-H12	Biolegend	105320
Brilliant Violet 605	Mouse CD90.2 (Thy1.2)	53-2.1	Biolegend	140317
APC/Cy7/ Alexa Fluor 700	Mouse/ human CD116	M1/70	Biolegend	101226/ 101222
Alexa Fluor 488	Mouse CD107a	1D4B	Biolegend	121608
PE	Mouse CD127 (IL-7Ra)	A7R34	Tonbo Biosciences	50-1271-U025
Alexa Fluor 647	Mouse NK1.1 (CD161)	PK136	Biolegend	108730
RedFluor 710	Mouse NK1.1 (CD161)	PK136	Tonbo Biosciences	80-5941-U100
Purified	Mouse NK1.1 (CD161)	PK136	BioXcell	BE0036
PE/Dazzle 594	Mouse CD172a (SIRPα)	P84	Biolegend	144015
PE/Cy5	Mouse CD197 (CCR7)	4B12	Biolegend	120113
APC/ Brilliant Violet 421	Mouse CD274 (B7-H1, PD-L1)	10F.9G2	Biolegend	124312/ 124315
Brilliant Violet 785	Mouse CD279 (PD-1)	29F.1A12	Biolegend	135225
Brilliant Violet 605	Mouse CD366 (Tim-3)	RMT3-23	Biolegend	119721
Biotin	Mouse/ human Dectin-1	2A11	GeneTex	GTX43894
APC	Mouse F4/80	BM8.1	Tonbo Biosciences	20-4801
Alexa Fluor 647	Mouse/ human Granzyme B	GB11	Biolegend	515406
Brilliant Violet 510	Mouse I-A / I-E	M5/114.15.2	Biolegend	107635
APC	Mouse IFN-g	XMG1.2	Tonbo Biosciences	20-7311-U100
PE	Mouse IL-2	JES6-5H4	Tonbo Biosciences	50-7021-U100
Alexa Fluor 488	Mouse/human Ki-67	11F6	Biolegend	151204
Brilliant Violet 421/ 785	Mouse Ly-6C	HK1.4	Biolegend	128032/ 128041
PerCP-Cyanine 5.5	Mouse Ly-6G	1A8	Tonbo Biosciences	65-1276-U025
PE	Mouse iNOS	N/A	Cell Signaling	14792
PE/ PE-Dazzle 594	Mouse/ human T-bet	4B10	Biolegend	644810/ 644827
PE/Dazzle 594	Mouse TNF-α	MP6-XT22	Biolegend	506346
PE	Mouse / rat XCR1	ZET	Biolegend	148203
Biotin	Mouse / rat XCR1	ZET	Biolegend	148212
Brilliant Violet 605	Streptavidin	N/A	Biolegend	405229
FITC	Human CD83	HB15e	Biolegend	305305
APC	Human CD86	IT2.2	Biolegend	305412
PE	Human CD163	GHI/61	Biolegend	333606
PE	Human CD274	29E.2A3	Biolegend	329706
PE	Human HLA-DR	L243	Biolegend	307606
FITC	Human IFN-g	4S.B3	Biolegend	502506
PE	Human IL-4	8D4-8	Biolegend	500705
Purified	Mouse PD-1	RMP1-14	BioXcell	BP0146
Unconjugated	Mouse CD3	SP7	Abcam	ab16669
Unconjugated	Mouse CD8	4SM15	eBiocience	14-0808
Unconjugated	Mouse/human Granzyme B	N/A	Abcam	ab4059
Unconjugated	Mouse Ki67	SP6	Thermo Fisher	MAS-14520
N/A	HRP-goat anti-rabbit	N/A	Jackson Immunoresearch	111-035-144
N/A	HRP-goat anti-rat	N/A	Jackson Immunoresearch	112-035-167
Purified	Anti-Tyrp1 (Tyrosinase-related protein 75)	TA99	BioXcell	BE0151
Unconjugated	Anti-Tyrp1 (Tyrosinase-related protein 75)	EPR13063	Abcam	ab178676
Purified	Mouse CD8	L3	BioXcell	BE0061
Purified	anti-VEGFR2	DC101	BioXcell	BE0060
