# Supplementary material for: Imprime PGG Enhances Anti-Tumor Effects of Tumor-Targeting, Anti-Angiogenic, and Immune Checkpoint Inhibitor Antibodies
Source: Front Oncol. 2022 May 26;12:869078. doi: 10.3389/fonc.2022.869078 (PMC9178990; doi:10.3389/fonc.2022.869078)
Supplement: Supplementary file 2 [file DataSheet_2.pdf]

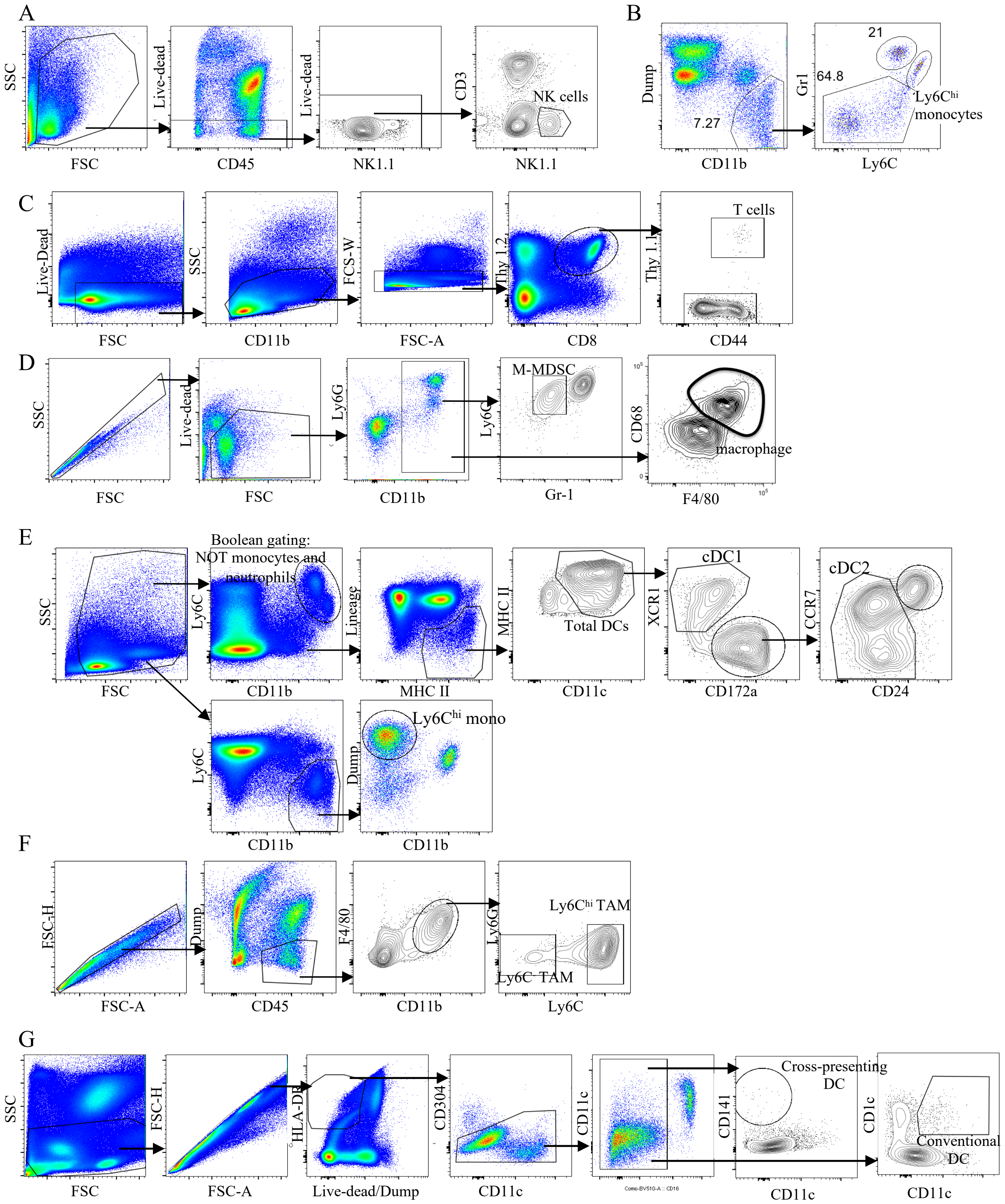

Supplementary Figure 1. Gating strategies for identification of immune cell subsets. Single cell suspensions of blood, sdLN, spleen and tumor were prepared, stained with cell-specific surface markers for identification and subsequently analyzed by flow cytometry as described in Materials and Methods. **(A)** Identification of NK cells from sdLN, **(B)** Ly6C^{hi} monocytes from mouse whole blood, **(C)** T cells from mouse whole blood and spleen, **(D)** m-MDSC and macrophages from spleen, **(E)** DC subsets and Ly6C^{hi} monocytes from sdLN, **(F)** Ly6C^{hi} & Ly6C^{-} TAM from mouse tumor, and **(G)** human DC subsets from whole blood are shown here.

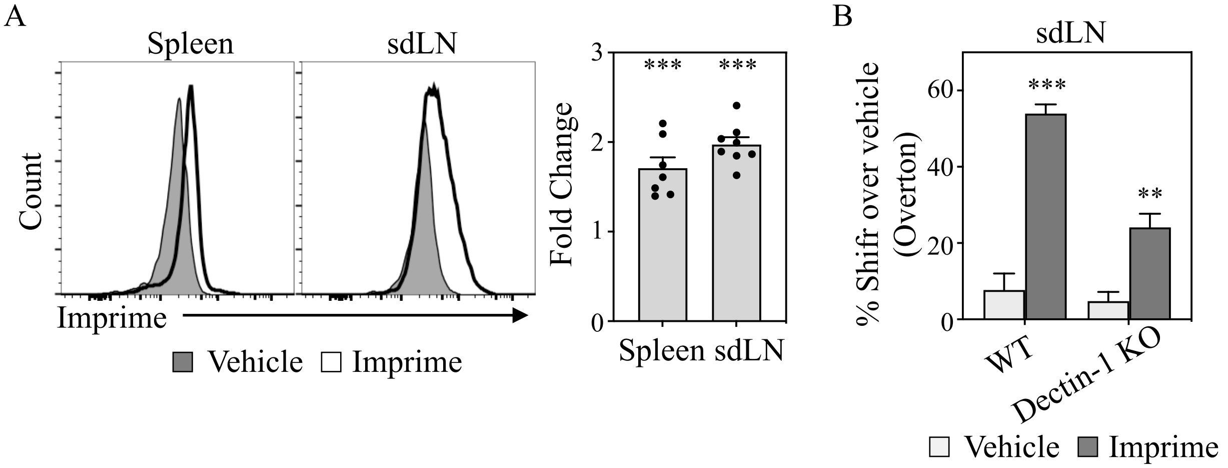

Supplementary Figure 2. Imprime binds Ly6C^{hi} monocytes in tumor-free mice in Dectin-1-dependent manner. Naïve or Dectin-1 KO C57BL/6 mice (n = 5-10) were injected i.v. with PBS or 1.2 mg Imprime. **(A)** Imprime binding of Ly6C^{hi} monocytes in spleen and sdLNs was analyzed by flow cytometry at 24 hours. Representative histogram plots from one mouse and mean fold increase of MFI over vehicle in spleen and sdLN are shown. **(B)** Overton method was used to normalize the % binding in sdLNs of WT and Dectin-1 KO mice. Representative results from ≥ 2 experiments are shown. All summary data are presented as mean ± SEM. Unpaired Student's *t* test was used to compare the changes to the vehicle group. *$p < 0.05$, **$p < 0.01$, ***$p < 0.001$.

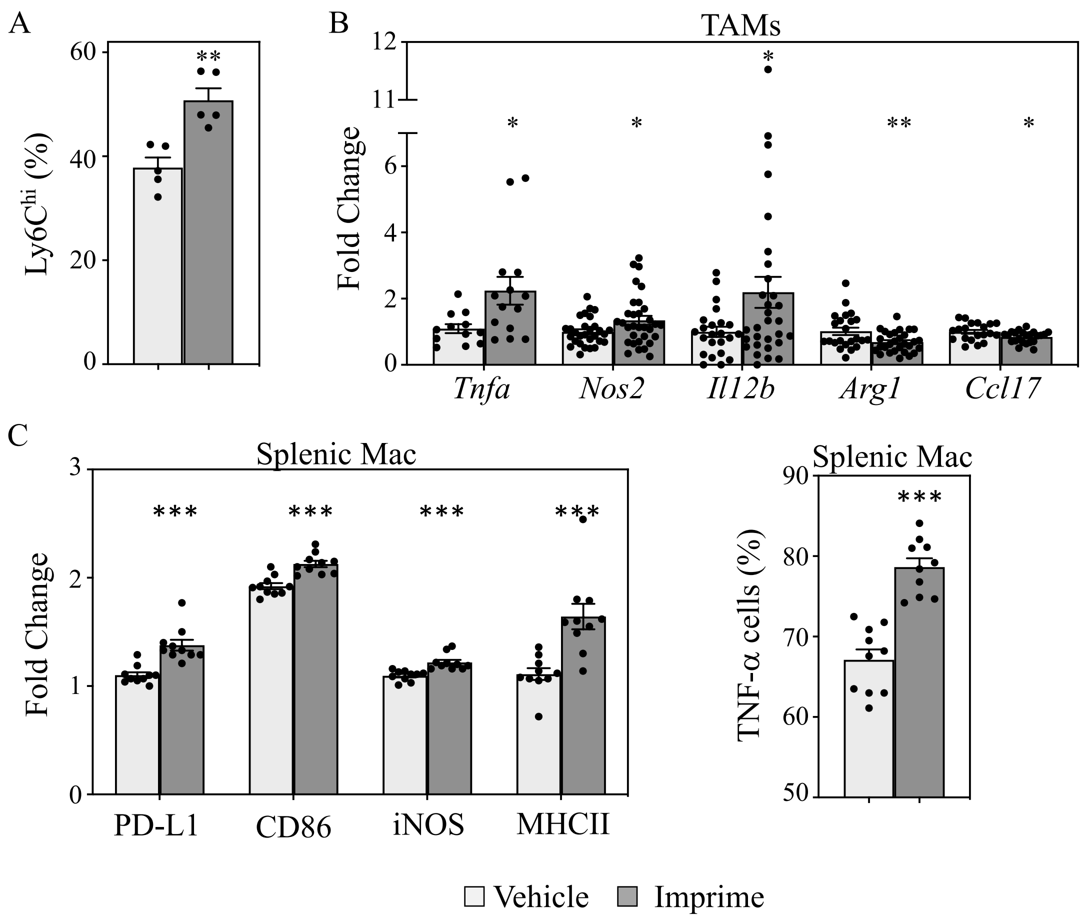

Supplementary Figure 3. Imprime modulates trafficking and activation of myeloid cells in the tumor microenvironment and secondary lymphoid organs in MC38 syngeneic mouse model. The MC38 syngeneic mouse model was established as described in Materials and Methods. Imprime or vehicle treatment started when tumors reached ~50mm^3 (n = 5-20/treatment group). **(A)** Percent of Ly6C^{hi} monocytes were determined by flow cytometry; (**B**) Gene expression analyses of M1/M2 markers were performed by bulk RNA analyses; **(C)** Activation markers and LPS-induced TNF-α production on splenic macrophages were analyzed by flow cytometry. All data are representative of at least 2 experiments and presented as mean ± SEM. Multiple *t* tests with Holm-Sidak's multiple comparison were used in **(B, C-left)** and Unpaired Student's *t* test was used in **(A, C-right)**. $^*p < 0.05$, $^{**}p < 0.01$, $^{***}p < 0.001$.

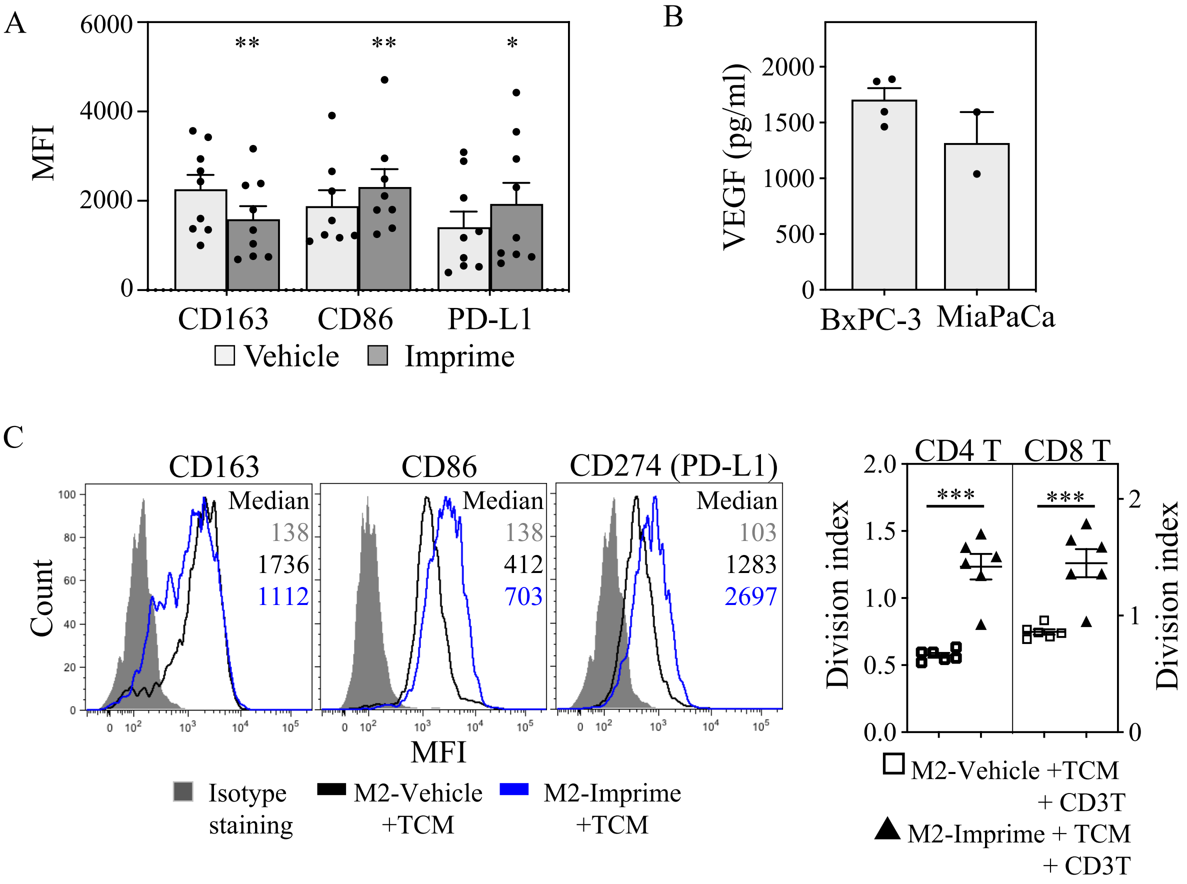

Supplementary Figure 4. Imprime activates M2 MDM even in the presence of TCM. M2-MDMs were prepared from monocytes as described in Materials and Methods. **(A)** Phenotype of vehicle- or Imprime-treated MDMs was assayed by flow cytometry. MFI of the activation markers of each individual donors shown. **(B)** VEGF level in the BxPC-3 and MiaPaCa culture supernatant was evaluated by ELISA. **(C)** MDMs were prepared in the presence of immunosuppressive cytokines containing TCM and evaluated for phenotype and ability to modulate CD3/CD28-stimulated T cell proliferation. At least 3 different human donors were tested and all summary data are presented as mean ± SEM. Unpaired Student's *t* test was used. *$p < 0.05$, **$p < 0.01$, ***$p < 0.001$.

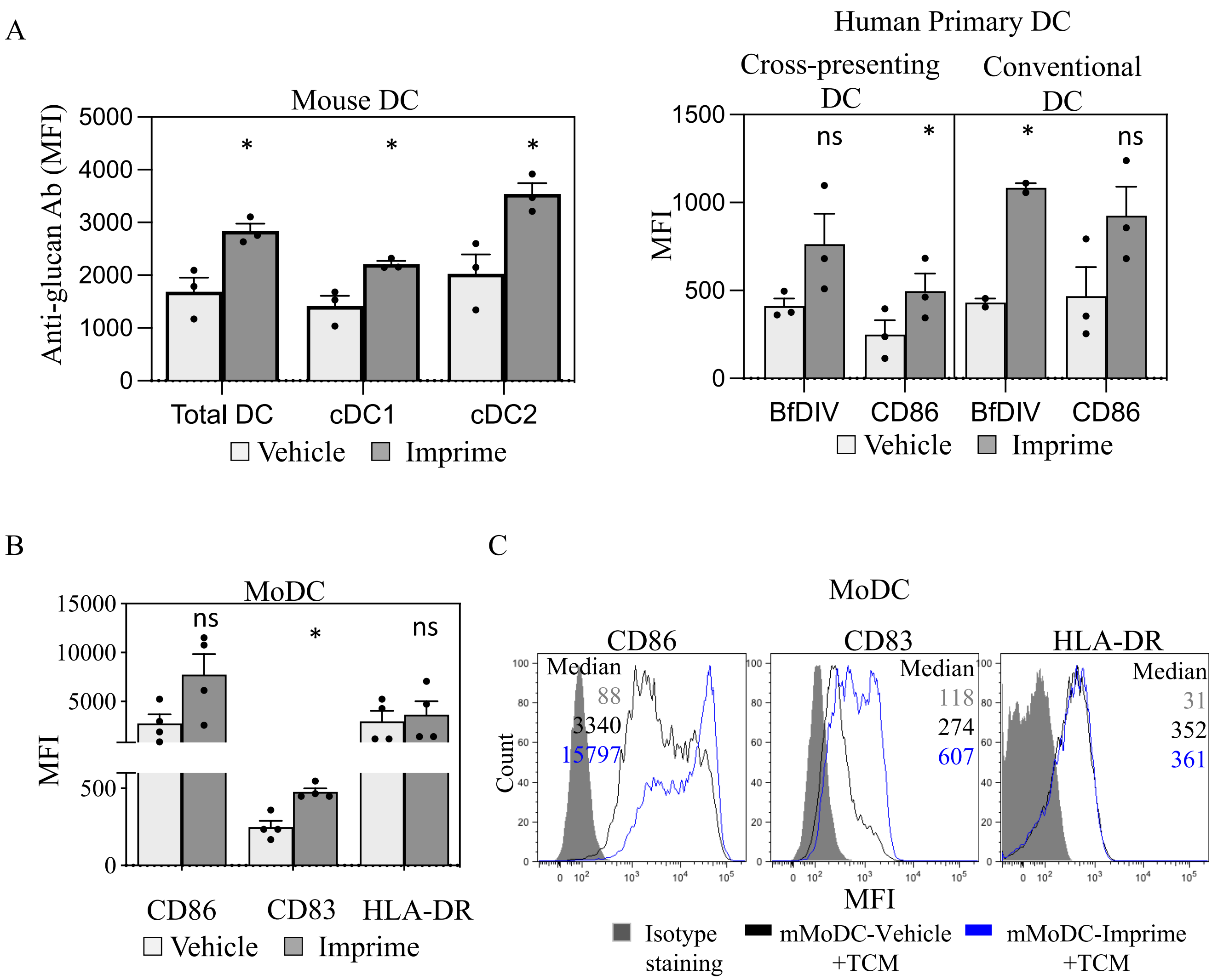

Supplementary Figure 5. Imprime binds and activates mouse, human primary DC and MoDC. **(A)** C57BL/6 mice (n = 3-5) were treated with Imprime or vehicle. 24 hours post administration, Imprime binding of mouse total DC, cDC1, and cDC2 was detected with an anti-glucan rabbit polyclonal primary antibody and analyzed by flow cytometry. Increased MFI of anti-glucan staining on Imprime-bound DC from each individual mouse is shown on the left. Imprime binding as detected with anti-glucan Ab BfDIV and increased expression of CD86 on human primary DC from different donors are shown on the right. **(B)** Increased expression of maturation and activation markers, CD83, CD86, and HLA-DR on MoDC from different donors measured by flow cytometry are shown. **(C)** Activated phenotype of Imprime-treated MoDCs prepared in the presence of BxPC3-TCM is shown. At least 3 different human donors were tested for both human primary DC and MoDC. All summary data are presented as mean ± SEM. Unpaired Student's *t* test was used. *p < 0.05, **p < 0.01, ***p < 0.001.

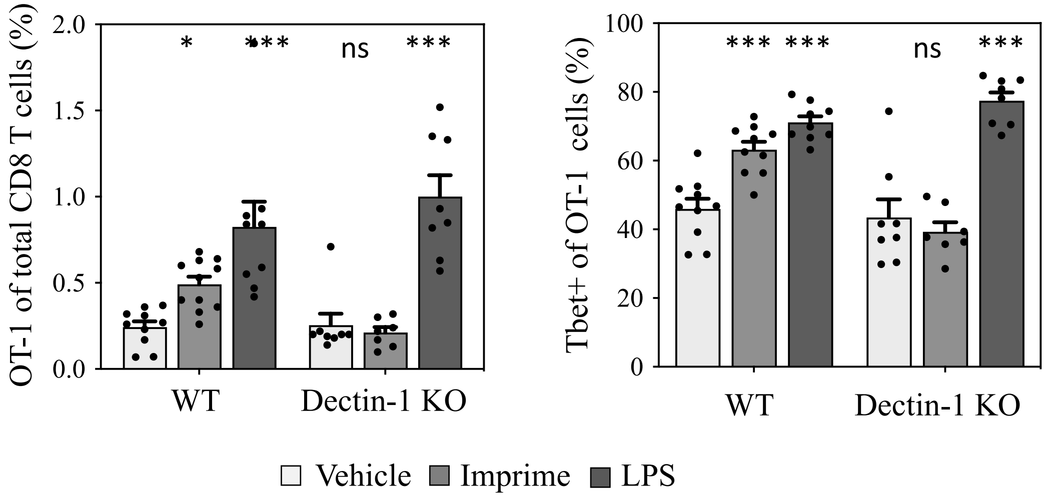

Supplementary Figure 6. Imprime drives antigen-specific CD8 T cell priming in a Dectin-1-dependent manner. Naïve phenotype CD44^{lo} OT-1 CD8 T cells were adoptively transferred into congenic WT or Dectin-1 KO recipient mice. Mice were then immunized i.v. with $OVA_{257\text{-}264}$/H-2K^{b} peptide and either vehicle, Imprime, or LPS. Percent OT-1 cells and percent Tbet^{+} of total CD8 T cells were compared in day 7 spleen of WT and Dectin-1 KO mice. The data shown here is a combination of at least 2 experiments and presented as mean ± SEM. 2-way ANOVA and Holm-Sidak multiple comparison was used. *$p < 0.05$, **$p < 0.01$, ***$p < 0.001$.

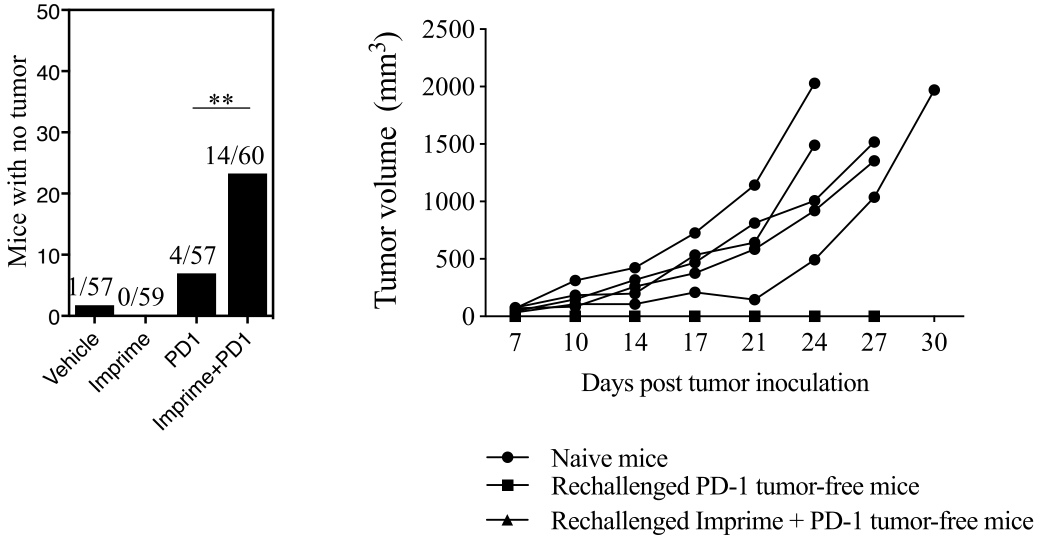

Supplemental Figure 7. Tumor clearance and development of memory response in more number of mice with Imprime and anti-PD1 Ab combination treatment. MC38 tumor model was established and treatments were administered as described in Materials and Methods. **(A)** Cumulative data from three experiments showing the number of mice that have complete responses in each of the treatment groups. **(B)** Tumor growth curves in 5 naïve mice and mice with complete responses in the anti-PD1 (2 mice) and Imprime + anti-PD1 group (7 mice) rechallenged with MC38 tumor implantation in the opposite flank are shown. Z-score at 95% confidence was calculated between the anti-PD-1 and anti-PD-1 + Imprime groups. $^{*}p < 0.05$, $^{**}p < 0.01$, $^{***}p < 0.001$.
